# A Framework for the Establishment of a Cnidarian Gene Regulatory Network for “Endomesoderm” Specification: The Inputs of ß-Catenin/TCF Signaling

**DOI:** 10.1371/journal.pgen.1003164

**Published:** 2012-12-27

**Authors:** Eric Röttinger, Paul Dahlin, Mark Q. Martindale

**Affiliations:** Kewalo Marine Laboratory, Pacific Biosciences Research Center, University of Hawai'i, Honolulu, Hawai'i, United States of America; University of Pennsylvania School of Medicine, United States of America

## Abstract

Understanding the functional relationship between intracellular factors and extracellular signals is required for reconstructing gene regulatory networks (GRN) involved in complex biological processes. One of the best-studied bilaterian GRNs describes endomesoderm specification and predicts that both mesoderm and endoderm arose from a common GRN early in animal evolution. Compelling molecular, genomic, developmental, and evolutionary evidence supports the hypothesis that the bifunctional gastrodermis of the cnidarian-bilaterian ancestor is derived from the same evolutionary precursor of both endodermal and mesodermal germ layers in all other triploblastic bilaterian animals. We have begun to establish the framework of a provisional cnidarian “endomesodermal” gene regulatory network in the sea anemone, *Nematostella vectensis*, by using a genome-wide microarray analysis on embryos in which the canonical Wnt/ß-catenin pathway was ectopically targeted for activation by two distinct pharmaceutical agents (lithium chloride and 1-azakenpaullone) to identify potential targets of endomesoderm specification. We characterized 51 endomesodermally expressed transcription factors and signaling molecule genes (including 18 newly identified) with fine-scale temporal (qPCR) and spatial (*in situ*) analysis to define distinct co-expression domains within the animal plate of the embryo and clustered genes based on their earliest zygotic expression. Finally, we determined the input of the canonical Wnt/ß-catenin pathway into the cnidarian endomesodermal GRN using morpholino and mRNA overexpression experiments to show that NvTcf/canonical Wnt signaling is required to pattern both the future endomesodermal and ectodermal domains prior to gastrulation, and that both BMP and FGF (but not Notch) pathways play important roles in germ layer specification in this animal. We show both evolutionary conserved as well as profound differences in endomesodermal GRN structure compared to bilaterians that may provide fundamental insight into how GRN subcircuits have been adopted, rewired, or co-opted in various animal lineages that give rise to specialized endomesodermal cell types.

## Author Summary

Cnidarians (anemones, corals, and “jellyfish”) are an animal group whose adults possess derivatives of only two germ layers: ectoderm and a bifunctional (absorptive and contractile) gastrodermal (gut) layer. Cnidarians are the closest living relatives to bilaterally symmetrical animals that possess all three germ layers (ecto, meso, and endoderm); and compelling molecular, genomic, developmental, and evolutionary evidence exists to demonstrate that the cnidarian gastrodermis is evolutionarily related to both endodermal and mesodermal germ layers in all other triploblastic bilaterian animals. Little is known about endomesoderm specification in cnidarians. In this study, we constructed the framework of a cnidarian endomesodermal gene regulatory network in the sea anemone, *Nematostella vectensis*, using a combination of experimental approaches. We identified and characterized by both qPCR and *in situ* hybridization 51 genes expressed in defined domains within the presumptive endomesoderm. In addition, we functionally demonstrate that Wnt/Tcf signaling is crucial for regionalized expression of a defined subset of these genes prior to gut formation and endomesoderm maintenance. Our results support the idea of an ancient gene regulatory network underlying endomesoderm specification that involves inputs from multiple signaling pathways (Wnt, FGF, BMP, but not Notch) early in development, that are temporarily uncoupled in bilaterian animals.

## Introduction

During metazoan development one cell gives rise to thousands of daughter cells, each acquiring a particular fate depending on their temporal and spatial coordinates within the organism. The information required to assume a specific fate of a given cell is present in the genome of all cells, requiring a fine tuned mechanism for controlling and coordinating gene expression during development of the growing embryo. The fate of each cell is determined by its set of expressed genes and controlled by the action of transcriptional activators and/or repressors whose activity is governed by intracellular (e.g. localized cytoplasmic factors, RNA binding proteins), or extracellular signals (e.g. endocrine or exocrine signaling pathways). All together, these components form gene regulatory networks that underlie the formation of distinct cell types or germ layers. Understanding the relationship between intracellular factors and extracellular signals can provide key insight in how and when the molecular and morphological characters of each organism are built.

Triploblastic organisms, also called “bilaterians” due to their bilaterally symmetrical body (possessing an anterior-posterior axis and dorso-ventral polarity), constitute the vast majority of all metazoan animals. These animals are characterized by the formation of three distinct primary germ layers during embryogenesis called the endo-, meso- and the ectoderm, that subsequently differentiate into more specialized adult tissues. Ectoderm gives rise to skin and nervous system, endoderm gives rise to the derivatives of the digestive tract including the intestine and digestive glands, and mesodermal derivatives include muscle, connective tissue, blood, coelomic cavities, kidneys/nephridia, somatic portions of the gonad, and skeletal elements. Both classic descriptions as well as modern molecular analyses of germ layer formation in bilaterian organisms as diverse as nematodes, sea urchins, and vertebrates have indicated that these decisions are largely made in a two steps: ectodermal fates first separate from a bicompetent endomesodermal (also called mesendodermal) domain, and then endodermal fates become distinct from mesodermal tissues [1]–[3].

In 2002, the extensive amount of experimental data collected during the past decades by the sea urchin community was assembled into a provisional endomesodermal (EM) gene regulatory network representing interactions between signals/transcription factors (TF) and their downstream targets, which in turn activate/repress other signals/TF's required for endomesoderm formation in the sea urchin embryo [4]–[11]. To date, a very limited number of model organisms have been used to establish GRN's for endomesoderm specification and/or differentiation (for review see [12]). Endomesodermal GRNs have been established only for the nematode *C. elegans* [13], the sea urchin (*S. purpuratus*, *P. lividus*, *L. variegatus*) [6], [7], [10], [14]–[16], a sea star (*A. miniata*) [17], [18] and *Xenopus* [19]. Comparison of the sea star and sea urchin endomesoderm GRNs indicates that there is a set of highly conserved genes, thought to be part of the “kernel” of the endomesodermal circuit present in the echinoderm ancestor [18], [20]. In *Drosophila*, a well-established genetic model system, mesoderm and endoderm are created by fundamentally different regions of the animal [21]–[23], reviewed in [24]. Although some of the endomesodermal kernel genes appear to be involved in gut formation in insects, the differences in gut development in flies has so far made it difficult to compare with other endomesodermal GRNs from other bilaterian studied.

The origin of the mesodermal germ layer and all of its unique cell types (e.g. muscle, connective tissue, blood, kidney and somatic gonad) during metazoan evolution is a matter of intense debate and investigation (reviewed in [25]–[34]. The sister group to all triploblastic animals is a group of animals called cnidarians (sea anemones, corals, sea fans, and ‘jellyfish’). Cnidarians are diploblastic animals formed exclusively by an epidermis (ectoderm) and a gastrodermis (also historically called entoderm). There are no classical bilaterian muscle cells [35] or a mesodermal tissue layer in cnidarians, however, the cnidarian gastrodermis is a bifunctional tissue capable of both absorption and contractile functions via myoepithelial cells [29], [36]–[38]. The cnidarian gastrodermis also express a large number of both endodermal factors and genes historically associated with mesoderm formation such as *otx*, *snail*, *twist* [26], [39], [40] suggesting that the cnidarian gastrodermis has a bifunctional endomesodermal capacity that never segregates into two distinct tissues. It also suggests that it contains components of an ancestral triploblastic (bilaterian) endomesodermal gene regulatory network and that endodermal and mesodermal tissues in triploblastic organism may be derived from the bifunctional gastrodermis of the cnidarian/bilaterian ancestor. This provides us with the opportunity to gain insight in to the ancestral endomesodermal GRN in a living organism.

Recent studies have shown the favorable features and utility of the cnidarian *Nematostella vectensis* as a developmental and evolutionary model system [39], [41]–[46]. Importantly the whole genome has been recently sequenced by the Joint Genome Institute (JGI) and is publicly available [47]. As an anthozoan, it has a simple anatomy, an undetermined long life span, and a short life cycle of 10–14 weeks. The sexes are separate allowing *in vitro* fertilization and manipulating the light cycle can induce spawning of several hundreds of eggs/female. When raised at 17 degrees Celsius, a hollow blastula forms approximately 10–12 hours post fertilization (hpf) and the embryo begins to gastrulate around 24–28 hpf via invagination at the animal pole [48]–[50], the side of the animal that gives rise to the single oral opening and the gastrodermis (endomesoderm).

The canonical Wnt (cWnt) signaling pathway plays crucial roles during various bilaterian developmental processes such as axis specification and germ layer formation [51]–[58]. Recent studies have suggested that the cWnt/β-catenin pathway has an ancient role in axis and endomesoderm formation in *N. vectensis* [50], [59]. Treatments with lithium chloride (LiCl), perturbs nuclear ß-catenin (nß-catenin) distribution ectopically stabilizing nß-catenin in all blastomeres along the A/V axis and induces hyper-proliferation of endomesoderm. In addition, inhibition of the cWnt pathway by overexpressing either cadherin, a cell adhesion molecule that titrates the cytoplasmic pool of ß-catenin, or a β-catenin∶engrailed fusion (acting as transcriptional repressor) blocks gastrulation and endomesoderm formation [59]. Recently, Lee and colleagues have shown that Dsh is required for nuclearization of β-catenin and endomesoderm development by over expression of a dominant negative form of Dsh (NvDsh-DIX) that specifically stabilizes the canonical Wnt pathway [50]. Taken together, those results show that the cWnt/β-catenin pathway is required for proper endomesoderm formation in *N. vectensis*. Although the authors of these studies suggest that endoderm specification may be affected by cWnt inhibition, they only characterize endomesodermal gene expression by the analysis of a single gene at the late gastrula stage, a time point long after endomesoderm specification. Therefore, additional information is required to better understand early endomesoderm specification in *N. vectensis*.

Deciphering the cnidarian endomesodermal GRN is important for a number of reasons. It can become a useful resource to understand the basic developmental mechanisms of a “simple” animal, help understand germ layer formation in a diploblastic animal providing a framework for future developmental studies (predicting relationships with new identified genes, *cis*-regulatory analysis etc.), and comparative work may provide important information to understand how components of the GRN have been adopted, re-wired or co-opted that lead to the evolution of biological novelties (such as “true” mesoderm). Recent studies comparing echinoderm endomesodermal (EM) GRNs, revealed changes in GRN structure and offered the opportunity to present testable hypotheses for the molecular basis of body plan and cell type evolution across echinoderms [17].

In order to understand how and when the cnidarian endomesodermal GRN is deployed and to define the initial input of the cWnt pathway, we employed a set of complementary approaches (Figure S1). We re-analyzed previously published genes expressed in the pharynx or gastrodermis using a combination of fine scale qPCR for the first 48 hours of development coupled to whole mount *in situ* hybridization prior to the onset of gastrulation. In order to identify additional putative members of the cnidarian “endomesoderm” GRN, we performed genome wide microarrays on mRNA extracted from embryos in which the canonical Wnt pathway has been activated using two distinct reagents, Lithium chloride (LiCl) and 1-azakenpaullone (AZ). These two pharmaceutical drugs both induce ectopic nuclearization of ß-catenin, but intriguingly, cause significant differences at the molecular and morphological levels. Fine scale temporal and spatial gene expression analysis of newly identified genes in combination with re-evaluated expression data allowed us to draw a first blueprint of putative transcriptional interaction in the presumptive cnidarian endomesoderm (gastrodermis). Finally, using complementary knockdown experiments, we investigated the earliest input of the cWnt pathway into the first non-bilaterian endomesoderm GRN. While inhibition of cWnt blocks pharynx formation, affects endomesodermal gene transcription and is required for spatial restriction of gene expression domains within the animal hemisphere prior to gastrulation, our global analysis suggests that proper specification of endomesoderm in *N. vectensis* also requires activation of both FGF and BMP, but not Notch, signaling pathways.

## Results

### Ectopic activation of the canonical Wnt pathway using two distinct Gsk3ß inhibitors (LiCl or 1-azakenpaullone) induces different phenotypes

**Figure 1:**
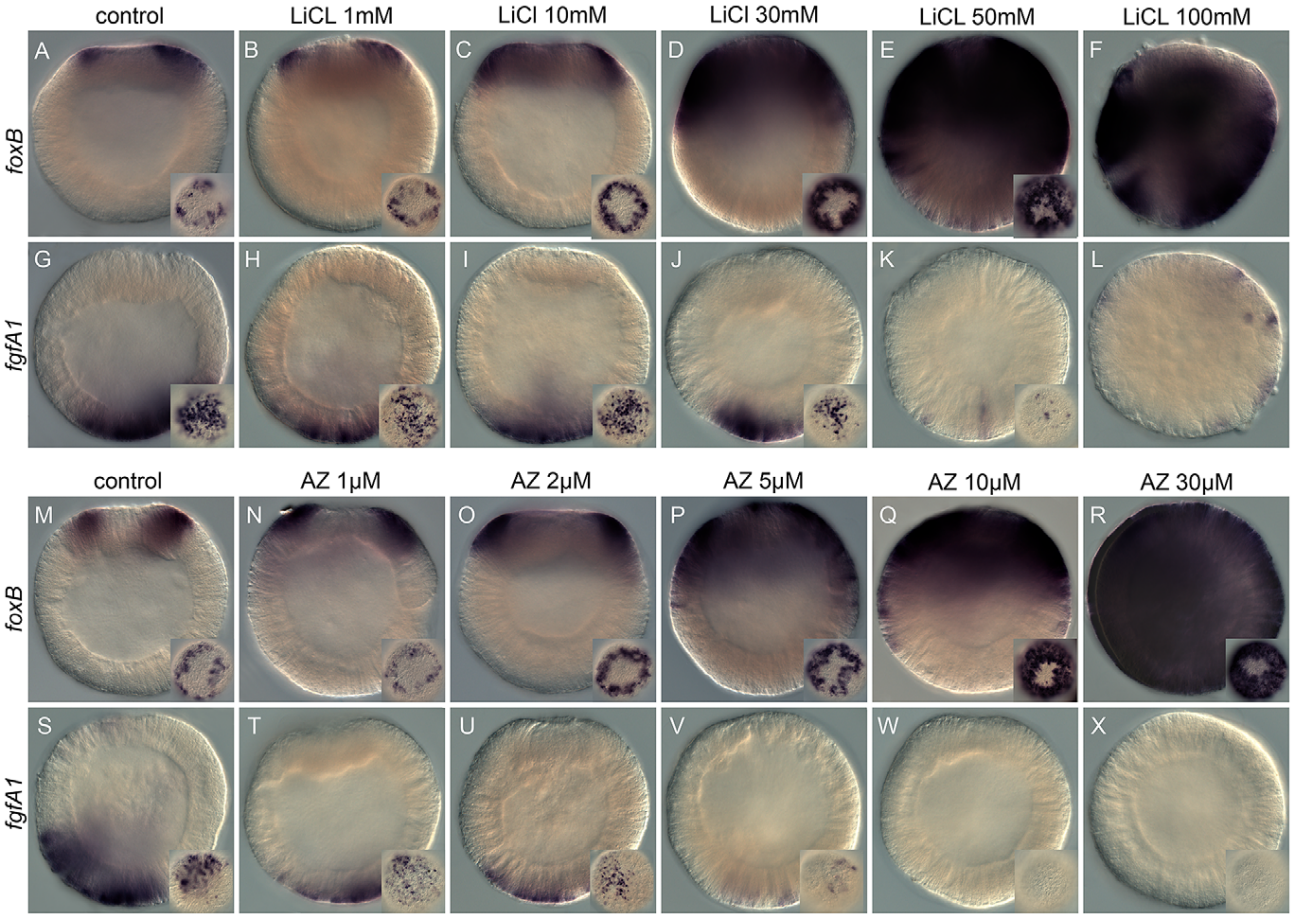
Dose-dependent effects of the Gsk3ß inhibitors, lithium chloride (LiCl), and 1-azakenpaullone (AZ) on embryonic gene expression. Control blastula stages at 24 hpf (A,G,M,S) and embryos treated with increasing concentrations of LiCl (B–F, H–L) or AZ (N–R,T–X). *In situ* hybridization on blastula stages using *NvfoxB* (A–F, M–R) or *NvfgfA1* (H–L, T–X) antisense probes. All images are lateral views with the presumptive endomesoderm (animal pole, future oral pole) to the top. The insets correspond to animal pole views.

Activation of the cWnt pathway can be induced by inhibition of Gsk3ß using pharmaceutical or chemical components. In order to compare the concentration dependent effects of two Gsk3ß inhibitors, lithium chloride (LiCl) and 1-azakenpaullone (AZ) we treated zygotes with increasing concentrations of LiCl and AZ and analyzed their effects on expression of *NvfoxB* (an oral/pharyngeal marker [42]) in the presumptive oral endomesoderm) and *NvfgfA1* (an aboral pole marker [60], [61]) at 24 hpf, prior to the onset of gastrulation and the appearance of endomesoderm (Figure 1, Table 1).

**Table 1:** Dose dependent effects of LiCl and AZ on *Nv-foxB* and *Nv-fgfA1* expression.

	Ctrl	Az 1 µM	Az 2 µM	Az 5 µM	Az 10 µM	Az 30 µM	LiCl 1 mM	LiCl 10 m	LiCl 30 mM	LiCl 50 mM	LiCl 100 mM	
***foxB***	108	89	93	46	9		76	56	12		*	wild type expression
		5	9	53	53	4	2	13	67	7	*	expanded
				2	26	15			7	53	*	strongly expanded (∼1/2 embryo)
					3	42			2	23	*	entire embryo
	**108**	**94**	**102**	**101**	**91**	**61**	**78**	**69**	**88**	**83**	**/**	**total**
***fgfA1***	113	58	64	2	2	2	48	52	7	6	*	wild type expression
	12	5	9	46	13	4	9	14	42	28	*	reduced
	4	2	7	13	63	76	3	5	8	33	*	absent
	**129**	**65**	**80**	**61**	**78**	**82**	**60**	**71**	**57**	**67**	**/**	**total**

Dose-dependent effects of LiCl and AZ analyzed by *in situ* hybridization. Analyzed AZ or LiCl concentration as indicated in Row 1 (light green) and number of embryos with phenotype scored based on expansion/reduction of the domain of expression as indicated in the column on the right. (*) under LiCl indicate a developmental delay/toxicity at that concentration.

With the exception of embryos treated with 100 mM LiCl that appeared developmentally delayed (Figure 1F, 1L), the general external morphology of the AZ and LiCl treated embryos (Figure 1B–1E, 1H–1K, 1N–1R, 1T–1X) resembled blastula control embryos (Figure 1A, 1G, 1M, 1S). Both treatments induced in a concentration dependent manner an extension of *NvfoxB* expression towards the vegetal hemisphere (Figure 1B–1E, 1N–1R) and a decrease in *Nvfgfa1* expression (Figure 1H–1K, 1T–1X), compared to control embryos (Figure 1A, 1G, 1M, 1S). However, while *Nv-fgfA1* expression was undetectable in AZ treated embryos at 10 µM and 30 µM (Figure 1W, 1X) its expression appeared only slightly reduced in LiCl treated embryos at the highest concentrations (Figure 1J, 1K). Based on the strong expansion of *Nv-foxB* expression in 30 mM LiCl and 10 µM AZ treatments (Figure 1D and 1Q, Table 1) we utilized these treatments for further developmental and molecular characterization.

**Figure 2:**
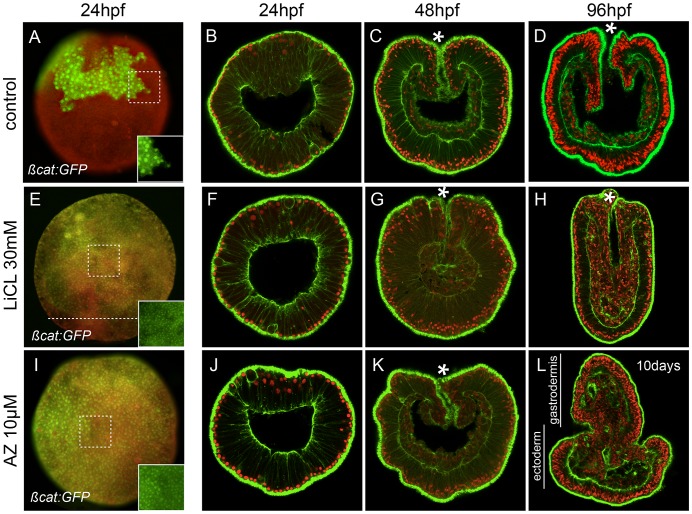
Ectopic activation of canonical Wnt signaling. (A–D) Control, (E–H) lithium chloride (LiCl) treated or (I–L) 1-azakenpaullone (AZ) treated embryos. (A,E,I) Embryos injected with mRNA encoding Nvß-catenin:GFP. The insets correspond to the details of the dashed squares to show the green nuclear ßcatenin (nß-catenin) localization in ectopic domains. Red (rhodamine) dextran was co-injected with *Nvß-catenin:GFP* mRNA and the merged images are shown in (A,E,I, see Figure S2 for the individual images). The dashed line in E indicates the absence of nß-catenin at the vegetal pole. Confocal z-sections using phalloidin (green) to show f-actin filaments and propidium iodide (red) to visualize the nuclei. (B,F,J) blastula stages (24 hpf), (C,G,K) late gastrula stages (48 hpf), (D,H) early planula (96 hpf) or (L) 10 day old planula larvae (see Figure S3 for better temporal resolution of the AZ phenotype). (hpf) hours post fertilization. All images are lateral views with the animal/oral pole (indicated by *) to the top.

To compare the effects of LiCl and AZ on ß-catenin nuclearization in *N. vectensis*, we injected mRNA encoding a GFP tagged form of Nvß-catenin (Nvßcat:GFP) [59] (Figure 2A), treated the injected uncleaved zygotes with either LiCl (30 mM, Figure 2E) or 1-azakenpaullone (10 µM, Figure 2I) and determined nuclear localization of ß-catenin at 24 hpf. As previously described [59], Nvßcat:GFP was uniformly expressed during early cleavage stages (data not shown), then progressively degraded in one hemisphere of the embryo and localized to the nuclei of cells in the presumptive endomesoderm (animal pole) prior to the onset of gastrulation (Figure 2A, Figure S2 [59]). In both treatments (Figure 2E, 2I), the domain of nuclear localization of Nvßcat:GFP was drastically expanded compared to control embryos. However, in LiCl treated embryos the nuclear localization of ß-catenin did not appear to extend all the way to the vegetal pole (aboral pole, Figure 2E), while in AZ treated blastula stages all cells of the embryo showed nuclear staining (Figure 2I).

Treatment of embryos with either LiCl or AZ did not cause any visible developmental perturbation for the first 48 hours post fertilization and the embryos gastrulated normally (Figure 2B, 2C, 2F, 2G, 2J, 2K). However after four days of development when control embryos reached the planula stage (Figure 2D), we distinguished two clear phenotypes resulting from the treatments. LiCl treated embryos became elongated with an increased amount of disorganized endomesodermal tissue and were devoid of any definite pharyngeal structure (Figure 2H, [59]). In contrast, AZ treated embryos displayed presumptive pharyngeal structures and endomesoderm everting from the oral pole, causing progressive exogastrulation after 10 days of development (Figure 2L, Figure S3). In AZ treated embryos the formation of endomesoderm increased at the expense of ectodermal tissue. The extension of *Nv-foxB* expression and nuclear ß-catenin localization towards the vegetal pole suggests a shift of the endomesoderm-ectoderm boundary and may involve changes in proliferation rates of endomesodermal cells (Figure 2L). Both of these treatments reinforce the idea that interfering with cWnt signaling affects endomesoderm formation in *N. vectensis* development. However, the distinct phenotypes suggested differences in either the efficacy or specificity of drug interaction.

Taken together these results support previous ideas of an ancestral role of Wnt/ß-catenin in endomesoderm specification and axial patterning in *N. vectensis* [50], [59] and suggest that AZ might be more effective than lithium in affecting the cWnt pathway.

### LiCl and AZ treatments affect surprisingly different sets of downstream targets

**Figure 3:**
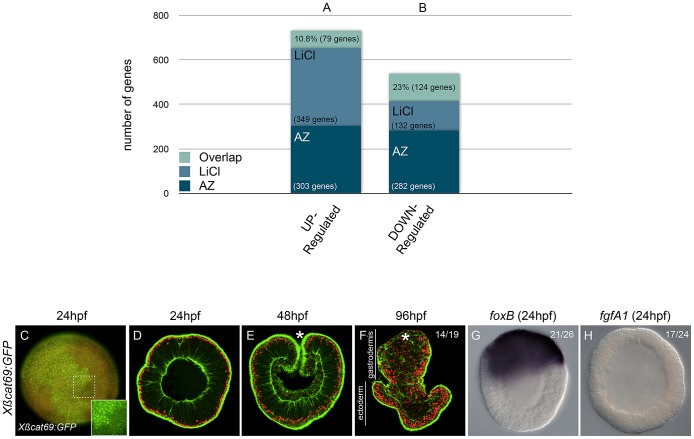
The effects of AZ and LiCl on global gene expression and their specificity in activating canonical Wnt signaling. (A,B) Predicted genome-wide microarray comparison of the effects of LiCl or AZ treatments. Genes that were significantly (P<0.05) at least 2-fold up- or downregulated were included in this analysis (C–H) Embryos injected with mRNA encoding a stabilized form of ß-catenin (Xßcat69:GFP). Red (rhodamine) dextran was coinjected with the mRNA (C). The inset in C corresponds to the detail of the dashed square to show nß-catenin localization in ectopic domains. Confocal z-sections using phalloidin (green) to stain f-actin filaments and propidium iodide (red) to visualize the nuclei in embryos of indicated stages (D–F). (C,D) blastula stages (24 hpf), (E) late gastrula stages (48 hpf), (F) planula larvae (96 hpf). *In situ* hybridization on *Xßcat69:GFP* injected blastula stages using (G) *NvfoxB* or (H) *NvfgfA1* antisense probes. Controls are the same as in Figure 1 and Figure 2 and all images are lateral views with the animal/oral pole (indicated by *) to the top. (hpf) hours post fertilization. Ratios in F,G,H indicate the number of embryos displaying the phenotype shown in the image to the total number of analyzed embryos.

In order to identify genes expressed in the presumptive endomesoderm of *N. vectensis*, and to analyze in more detail the similarities (and differences) in Gsk3ß inhibition using different reagents, we treated zygotes with either AZ or LiCl, extracted RNA prior to the onset of gastrulation (24 hpf) and screened an expression array designed to represent all protein coding genes in the *N. vectensis* genome. Out of 24,021 represented genes in our Nimblegen (Inc.) expression microarray, we selected genes with a significant 2-fold or greater change compared to the wild-type controls in the average of two biological replicates. Although the Pearson's correlation factors between biological replicates were low (0.53 and 0.42 for the AZ and LiCl arrays respectively), a total of 399 or 411 genes were significantly (P<0.05) upregulated in AZ or LiCl treated embryos, respectively, while 362 or 256 genes were significantly (P<0.05) down regulated in AZ or LiCl treated embryos, respectively (Table S1). To gain insight into the percentage of genes that are affected by either one of the cWnt activating treatments, we compared the two datasets to determine the degree of overlap of significantly up- or downregulated genes (Figure 3A, 3B).

Surprisingly, from the total of 731 unique significantly upregulated genes, only 79 genes (10.8%) were shared in both datasets. Of the remaining 652 genes, 303 genes (41,5%) were upregulated by AZ but not by LiCl and 349 genes (47.7%) were upregulated by LiCl but not by AZ (Figure 3A). Similarly, from a total of 538 genes that were significantly downregulated in both treatments, 132 genes (25.7%) were unique to LiCl, 282 genes (52.4%) were unique to AZ and only 124 genes (23%) were shared between the two treatments (Figure 3B).

Both compounds are supposed to target the ATP-binding pocket of Gsk3ß [62] and have been used in a wide range of organisms to study the role of cWnt signaling during early development [55], [63]–[66], regeneration [67] and cells in culture [68], [69]. Previous biochemical studies have described the difference in Gsk3ß affinity of AZ and LiCl [62] and shown that lithium chloride has additional targets such as inositol-phosphate phosphatases [70]. In order to gain insight into which Gsk3ß-inhibiting treatment in *N. vectensis* may be more specific to cWnt activation we over-expressed a stabilized form of *Xenopus* ß-catenin-GFP (Xßcat69:GFP, [50], [59] in which the GSK-3ß/CK-1 phosphorylation sites had been mutated to alanines and is resistant to proteolytic destruction [71].

In contrast to LiCl, but similar to AZ treatments, over-expression of *Xßcat69:GFP* mRNA induced ectopic localization of its protein in the nuclei of all cells along the oral-aboral axis (Figure 3C) and caused a strong exogastrulation phenotype after 4 days of development (Figure 3D–3F). In addition, expression of *Nv-foxB* in *Xßcat69:GFP* mRNA injected embryos was strongly expanded (Figure 3G), and *Nv-fgfA1* expression downregulated (Figure 3H) similar to that seen in AZ treatments (Figure 1Q, 1W). These observations suggest that in *N. vectensis* the effects caused by AZ treatments may reflect a more specific activation of the cWnt pathway than LiCl, although a more thorough analysis perhaps including other commonly used Gsk3ß inhibitors such as alsterpaullone [72]–[75] is required to identify the best cWnt activator in this system.

### Identification of 104 genes encoding transcription factors and signaling molecules affected by ectopic cWnt activation

cWnt signaling has previously been shown to be involved in endomesoderm formation in *N. vectensis* [50], [59] and ectopic activation of the pathway not only induces exogastrulation (Figure 2L, Figure 3F) but also the expansion of at least one endomesodermal transcription factor in the animal hemisphere prior to the onset of gastrulation (Figure 1D and 1Q, Figure 3G). To determine additional transcriptional differences between nß-catenin stabilized and control embryos with the goal of identifying putative genes that are required for specification and formation of endomesoderm in *N. vectensis*, we used gene profiling with a *N. vectensis* specific oligonucleotide based genome-wide microarray (Nimblegen, Inc). We chose to analyze differential expression in late blastula stages prior to the onset of gastrulation (24 hpf) of AZ and LiCl treated embryos. Transcription factors and signaling molecules build the basis of complex gene regulatory network that are deployed during embryogenesis [14], [76]. Therefore, we focused on the identification and characterization of genes that can be separated in the following classes: i) transcription factors, ii) signaling molecules (ligands and receptors) and iii) signaling pathway modulators (extracellular, membrane bound or cytoplasmic), that will constitute the main structure of the cnidarian endomesoderm GRN. Although the specificity of LiCl to activate the canonical Wnt pathway is questionable, at least one gene expressed in the presumptive endomesoderm, *Nv-foxB*, was visibly upregulated in embryos treated with that chemical (Figure 1D). For the purpose of identifying the largest possible set of new genes putatively playing a role in the gene regulatory network underlying endomesoderm formation in *N. vectensis*, we included microarray data from LiCl as well as AZ treatments that displayed at least a 2-fold upregulation from two biological replicates (Table S1).

Of the 731 genes identified as being upregulated by LiCl or AZ treatments, 104 unique genes belonging to distinct definitive/putative transcription factors or signaling molecules (Table 2) met our selection criteria for detailed characterization.

**Table 2:** Selection of 104 genes upregulated after LiCl or AZ treatments.

SpotID	Gene Name/Best Blast Hit	Gene Bank Accession Number	AZ or LiCl	Published expression pattern	Publication
124360	NvActivin	(ABF61781.1)	**AZ (T)**	yes	Matus et al. 2006
87636	NvAdmp-related	**JQ959545** (NvCmp in Matus et al. 2006)	AZ (V)	-	
214439	NvAlk2-like	**JQ959546**	AZ (V)	-	
80987	NvAnthox9	(ACT36593.1|)	AZ (V)	-	Ryan et al. 2007
31519	NvAp2-like	**JQ959547**	**LiCl (T)**	-	
106438	NvAshB	(BAJ13484)	AZ (V)/LiCl DOWN (V)	yes	Simionato et al. 2007, Marlow et al. 2011, Layden et al.2011
248853	NvAxin1-like	**JQ959548**	LiCl (V)	-	
135081	NvBicaudalC-like1	**JQ959549**	**AZ (T)**	-	
135116	NvBicaudalC-like2	**JQ959550**	AZ (V)	-	
168498	NvBmp2/4	(AAR13362.1)	LiCl (V)	yes	Matus et al. 2006
200866	NvBmpR-like	**JQ959551**	AZ (V)/LiCl (T)	-	
770	NvBrachyury	(AAO27886.2)	AZ (V)	yes	Scholz et al. 2003
111628	NvCek3-like (FgfR-like gene)	**JQ959552**	**LiCl (T)**	-	
113172	NvDmbxC/NvK50-4	(ABG67868.1/ABB83746.1)	AZ (V)/LiCl (V)	-	Chourrout et al. 2006, Ryan et al 2006
48861	NvDmbxF/NvDmbxE/K50-3	(ABB83731.1/ABB83743.1/ABG67867.1)	**LiCl (T)**	-	Chourrout et al. 2006, Ryan et al 2006
150808	NvDuxB/NvDuxC/DuxA/Q50-8	(ABB83732.1/ABB83734.1/ABB83737.1/ABG67886.1)	**LiCl (T)**	-	Chourrout et al. 2006, Ryan et al 2006
47573	NvElkA-like	**JQ959553**	**AZ (T)/LiCl (T)**	-	
108513	NvEtsB-like	**JQ959554**	AZ (V)	-	
101680	NvEvx	(AAZ94820.1/AF020951_1)	AZ (V)	-	Finnerty et al. 1997
212165	NvFgf20-like	**JQ959555**, (Nv212165 in Matus et al. 2007)	AZ (V)/LiCl (V)	-	
98926	NvFgf8/17-like	**JQ959556** (Nv204532 in Matus et al. 2007)	**AZ (T)**	-	
25772	NvFgf8B	(ABN70837.1)	LiCl (V)	-	Matus et al. 2007
84228	NvFlamingo-like	**JQ959557**	AZ (V)	-	
141657	NvFollistatin-like	**JQ959558**	AZ (V)	-	
165261	NvFoxA	(AAS13442.1)	AZ (V)	yes	Magie et al. 2006
110212	NvFoxA/B-like	**JQ959559** (Nematostella_110212 in Sanatagata et al. 2012)	AZ (V)	-	
187332	NvFoxB	(ABA03229.1)	**AZ (T)**	yes	Magie et al. 2006
5001	NvFoxO1-like	**JQ959560** (Nematostella_5001 in Sanatagata et al. 2012)	AZ (V)/LiCl (V)	-	
38679	NvFoxO2-like	**JQ959561** (Nematostella_38679 in Sanatagata et al. 2012)	AZ (V)/LiCl (V)	-	
201028	NvFoxQ1	**JQ959562** (Nematostella_201028 in Sanatagata et al. 2012)	AZ (V)	-	
200356	NvFoxQ2-like	**JQ959563** (Nematostella_200356 in Sanatagata et al. 2012)	AZ (V)	-	
206468	NvHd007/NvIrx	(ABB83733.1/ABG67891.1)	**LiCl (T)**	-	Chourrout et al. 2006, Ryan et al 2006
6595	NvHD017/NK-like 11	(ABB86468.1/ABG67819.1)	**AZ (T)**	-	Chourrout et al. 2006, Ryan et al 2006
17677	NVHD023/NVHD076/NK-like 6	(ABB86473.1/ABB86492.1/ABG67814.1)	**AZ (T)/LiCl (T)**	-	Chourrout et al. 2006, Ryan et al 2006
48953	NvHd031/Q50-5	(ABB86429.1/ABG67831.1)	LiCl (V)	-	Chourrout et al. 2006, Ryan et al 2006
114232	NvHD032/NK-like 7	(ABB86430.1/ABG67815.1)	**AZ (T)/LiCl (V)**	-	Chourrout et al. 2006, Ryan et al 2006
47299	NvHD042/NK-like 8	(ABB86479.1/ABG67816.1)	AZ (V)/LiCl (V)	-	Chourrout et al. 2006, Ryan et al 2006
208353	NvHD043/NK-like 12	(ABB86432.1/ABG67820.1)	**AZ (T)/LiCl (T)**	-	Chourrout et al. 2006, Ryan et al 2006
57885	NvHD050/NK-like 17	(ABB86483.1/ABG67825.1)	AZ (V)/LiCl (V)	-	Chourrout et al. 2006, Ryan et al 2006
98238	NvHD056/NK-like 18	(ABB86438.1/ABG67826.1)	**AZ (T)**	-	Chourrout et al. 2006, Ryan et al 2006
47235	NvHD071/NK-like 5	(ABB86489.1/ABG67813.1)	**AZ (T)/LiCl (V)**	-	Chourrout et al. 2006, Ryan et al 2006
108663	NvHD102	(ABB86448.1)	**AZ (T)/LiCl (T)**	-	Ryan et al. 2006
69052	NvHD147/NK-like 9	(ABB86461.1/ABG67817.1)	**AZ (T)/LiCl (V)**	-	Chourrout et al. 2006, Ryan et al 2006
204200	NvHes3 (Hes1-like)	(JN982709.1)	**AZ (T)/LiCl (T)**	yes	Marlow et al. 2011
120428	NvHint3	(ABX89901.1)	AZ (V)/LiCl (T)	yes	Matus et al. 2008
80365	NvHlxA	(ABG67795.1)	AZ (V)/LiCl (V)	-	Chourrout et al. 2006
101731	NvHlxB9/NvMnx	(ABB86488.1/ABG67770.1)	AZ (V)	yes	Chourrout et al. 2006, Ryan et al 2006
129868	NvHlxG/NK-like 2	(ABB86478.1/ABG67810.1)	AZ (V)/LiCl (T)	-	Chourrout et al. 2006, Ryan et al 2006
101740	NvHox6	(AAD39348.1)	AZ (V)/LiCl (V)	-	Finnerty et al. 1998
197330	NvJumonji-like	**JQ959564**	LiCl (V)	-	
113057	NvK50-6	(ABG67870.1)	AZ (V)	-	Chourrout et al. 2006
95727	NvLmx	(DQ500873.1)	AZ (V)	yes	Chourrout et al. 2006, Srivastava et al. 2010
206032	NvMab21-like	**JQ959565**	LiCl (V)	-	
243969	NvMgf5-like	**JQ959566**	LiCl (V)	-	
128302	NvMox2	(AAP88428.2)	AZ (V)	-	Kwong et al. direct submission to GenBank
128289	NvMoxC	(AAZ94818.1)	**AZ (T)**	yes	Chourrout et al. 2006, Ryan et al 2006
128296	NvMoxD	(AAZ94819.1)	**AZ (T)**	yes	Chourrout et al. 2006, Ryan et al 2006
80927	NvMsxC	(ABG67794.1)	AZ (V)	yes	Ryan et al. 2007
80394	NvMsxLXb/NvMsxB	(ABB86439.1/ABG67793.1)	**AZ (T)**	-	Chourrout et al. 2006, Ryan et al 2006
222052	NvMyb-like	**JQ959567**	AZ (V)	-	
203298	NvMyophilin-like	**JQ959568**	**AZ (T)**	-	
245445	NvNkd1-like	**JQ959571**	**AZ (T)**	-	
92366	NvNfix-like	**JQ959569**	LiCl (V)	-	
247396	NvNgfR-like	**JQ959570**	LiCl (V)	-	
197288	NvNk-like 13	(ABG67821.1)	AZ (V)	-	Chourrout et al. 2006
59839	NvNk2E/NvNK2-like	(ABB86474.1/ABG67781.1)	LiCl (V)	-	Chourrout et al. 2006
214452	NvNscl2-like	**JQ959572**	LiCl (V)	-	
212484	NvOnecut-like	**JQ959573**	**AZ (T)**	-	
29762	NvParaxis-like	**JQ959574**	AZ (V)/LiCl (V)	-	
106504	NvPatched-like	**JQ959575**	AZ (V)	-	
112745	NvPax6-like	**JQ959576**	AZ (V)	-	
119149	NvPaxD2	(ABI30248.1)	AZ (V)/LiCl (T)	-	Matus et al. 2007
239957	NvPhtf1-like	**JQ959577**	AZ (V)	-	
51461	NvPorcupine-like	**JQ959578**	**AZ (T)**	-	
84135	NvRdsl3-like	**JQ959579**	AZ (V)	-	
11703	NvRdsl4-like	**JQ959580**	LiCl (V)	-	
110792	NvREPO/NvREVPOL	(ABB72471.1/ABG67880.1)	**AZ (T)**	yes	Marlow et al. 2009
210816	NvRet-like	**JQ959581**	**AZ (T)**	-	
37078	NvShavenbaby/ove-like	**JQ959582**	AZ (V)	-	
248037	NvSmad4-like	**JQ959583**	AZ (V)	-	
23431	NvSnip1-like	**JQ959584**	LiCl (V)	-	
239453	NvSos-like	**JQ959585**	AZ (V)/LiCl (V)	-	
201202	NvSprouty3-like	**JQ959586**	AZ (V)	-	
200081	NvTbx15-like	**JQ959587**	AZ (V)	-	
88753	NvTbx18-like	**JQ959588**	AZ (V)	-	
117456	NvTbx20-like	**JQ959589**	AZ (V)	-	
132332	NvTcf/Lef	(ABF55257.1)	AZ (V)	yes	Lee et al. 2008
151755	NvTgfbR-like	**JQ959590**	AZ (V)/LiCl (T)	-	
201970	NvTgfbR3-like	**JQ959591**	LiCl (V)	-	
234699	NvTwist	(AAQ23384.1)	AZ (V)	yes	Martindale et a. 2004
93991	NvUnc4	(ABB72463.1/ABG67875.1)	**AZ (T)**	-	Chourrout et al. 2006, Ryan et al 2006
120512	NvVasa-like	**JQ959592**	AZ (V)	-	
91847	NvVax/VAX	(ABB86441.1/ABG67801.1)	**LiCl (T)**	-	
241043	NvVcam1-like	**JQ959593**	LiCl (V)	-	
87486	NvVegfR-like	**JQ959594**	AZ (V)	-	
158342	NvWnt1	(AAT00640.1)	**AZ (T)**	yes	Kusserow et al. 2006
106241	NvWnt16	(ABF48091.1)	AZ (V)	yes	Lee et al. 2006
241352	NvWnt3	(ABF48092.1)	AZ (V)	yes	Lee et al. 2006
194914	NvWnt4	(AAV87174.1)	AZ (V)	yes	Kusserow et al. 2006
100329	NvWnt5	(AAW28133.1)	**AZ (T)/LiCl (T)**	yes	Kusserow et al. 2006
113133	NvWnt7-like	**JQ959595**	**AZ (T)**	-	
210076	NvWnt7b	(AAW28135.1)	**AZ (T)**	yes	Kusserow et al. 2006
115097	NvWnt8	(AAV64158.1)	**AZ (T)/LiCl (T)**	yes	Kusserow et al. 2006
91822	NvWntA	(AAT02182.1)	AZ (V)	yes	Kusserow et al. 2006
Legend:					
AZ		identified in 1-azakenpaullone array			
LiCL		identified in lithium chloride array			
(V)		variation, selected based on a 2-fold UP-regulation (not statistically significant)			
**(T)**		Ttest, selected based on a statistically significant UP-regulation			
LiCl (V) Down		Nv-BicaudalC-like1 is one of the two genes identified to be upregulated in AZ array, but downregulated in LiCl array (Figure S1).			

Selected transcription factors and signaling molecules that were significantly (P<0.05) at least 2-fold upregulated by AZ and LiCl treatments. SpotID: genome protein model ID (JGI) used for the array design. The gene name is based on the best blast hit (see Material and Methods) and if available the previously published name(s) is used. Color code and abbreviations are indicated in the table legend at the bottom.

The majority of the selected genes (∼66%, 64/104) belonged to various families of transcription factors (Table 2), defined by their structure and DNA binding motifs, and involved in diverse developmental and biological processes. The largest group of transcription factors we selected belongs to the homeodomain containing molecules (28/64, e.g *Nvevx*, *Nvhd050*, *NvhlxB9*) that constitute an ancient class of regulatory genes with diverse roles in fungi, plants and animals [77]. Other transcription factors that were upregulated following Gsk3ß inhibitor treatment prior to gastrulation in *N. vectensis* belong to the Forkhead (e.g *NvfoxQ1*, *NvfoxA*, *NvfoxB*), T-box (e.g *Nvtbx20-like*, *Nvbra*), Ets (e.g *NvelkA-like*), Mad1 (e.g *Nvsmad4-like*, *Nvnfix-like*), HMG (e.g *Nvtcf*), zinc finger (e.g *NvsnailA*), bHLH (e.g Nvtwist, *Nvhes3*) or achaete-scute (e.g *NvashB*). These data indicate that a diverse set of transcription factor families may be involved in endomesoderm formation during cnidarian development (Table 2).

The Wnt, Hedgehog (Hh), RTK (Receptor Tyrosine Kinase, e.g. FGFR), Notch, Tgfß/Activin and Bmp signaling pathways are associated with diverse biological events during embryonic development in metazoan and have been previously described from *N. vectensis* [50], [56], [59], [78], [79]. With the exception of Notch signaling, putative ligands and/or receptors associated with all remaining pathways have been upregulated by ectopic canonical Wnt activation (Table 2). In particular, we identified 9 of the 13 described *N. vectensis* Wnt ligands [56], [80], *Nvactivin* [81], three Activin/TGFß Receptor-like genes, *Nvbmp2/4* [81], *Nvadmp-related*, one Bmp Receptor-like gene, *Nvfgf8A* [61], two FGF-like, three Tyrosine Kinase Receptor-like genes, *Nvhint3* [82] and one Patched-like receptor gene (Table 2). Interestingly, we also identified *Nvfollistatin* [81] a putative modulator of Activin [83], *Nvsprouty3-like* a putative modulator of FGF [84], as well as three modulators of Wnt signaling, *Nvaxin-like*, *Nvnkd1-like* (naked cuticle) and *Nvporcupine-like* [85]–[87], suggesting that these three signaling pathways (Activin, BMP and FGF), in addition to cWnt signaling, are deployed to specify and pattern the early *N. vectensis* embryo.

53 of the 104 genes identified above have been previously isolated, however only 23 have had their expression pattern characterized (e.g. *Nvbrachyury*, *NvfoxA*, *Nvtcf/lef* [26], [39], [50], [88]). All but two (*Nvhint3* [82] and *Nvhes3* [79] of the 23 previously characterized genes are expressed in endomesodermally related regions during development, demonstrating the effectiveness of the approach in *N. vectensis*.

### Existence of at least four distinct co-expression domains within the animal hemisphere of *N. vectensis* embryos prior to the onset of gastrulation

**Figure 4:**
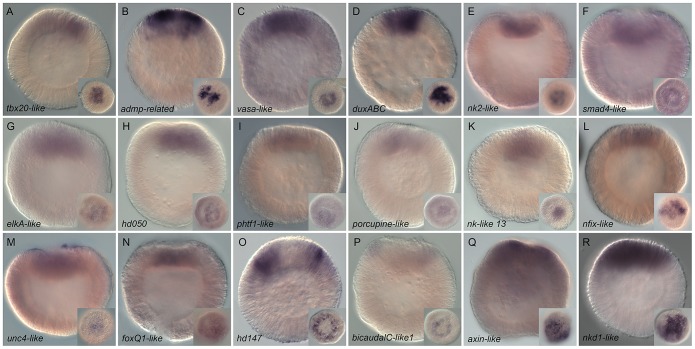
Spatial expression analysis on untreated embryos of 18 genes upregulated by LiCl or AZ treatments. Wild type gene expression analysis by *in situ* hybridization of genes upregulated by LiCl or AZ treatments. (A–R) All animals are blastula stages (24 hpf). All images are lateral views with the animal pole (presumptive endomesoderm) to the top and the insets correspond to animal pole views. Antisense probes used as indicated.

**Figure 5:**
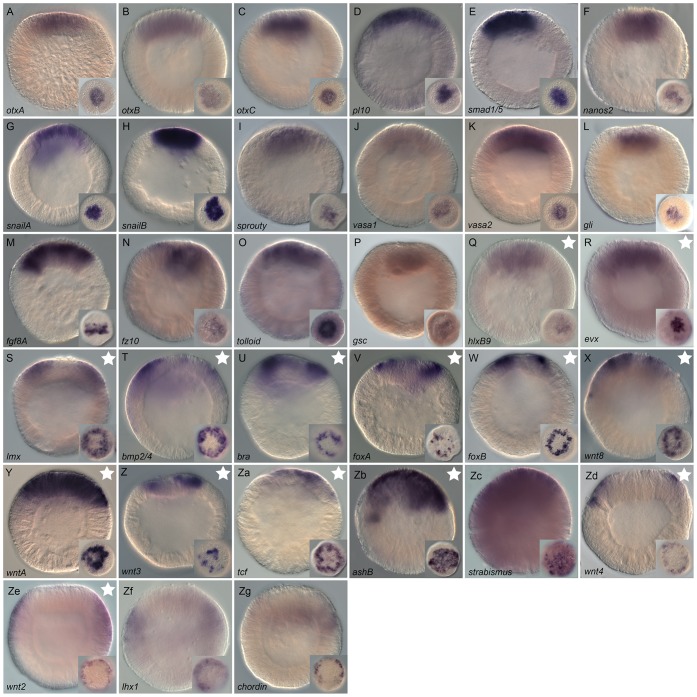
Gene expression re-analysis of previously published genes involved in endomesoderm development. Wild type gene expression analysis by *in situ* hybridization of previously published genes (for original publication, see Table S3). (A–Zg) All animals are blastula stages and the inset corresponds to animal views. Antisense probes used as indicated. All images are lateral views with the presumptive endomesoderm (future oral pole) to the top. The white stars (Q–Zb, Zd) indicate genes positively affected by LiCl or AZ treatments as determined by array experiments.

Previous work in *N. vectensis* has shown that there appears to be at least two distinct complementary expression domains within the animal plate that give rise to endomesdoerm prior to gastrulation: i) the central domain, located at the animal pole of the embryo and characterized by *NvsnailA* expression and ii) the central ring expressing *NvfoxA* that surrounds the central ring [26], [39], [48]. To gain a basic understanding of when and where the transcription factors and signaling molecules with potential roles in endomesoderm formation are expressed in the developing embryo, we performed whole mount *in situ* hybridization (Figure 4, Figure 5).

### Characterization of genes upregulated by LiCl and AZ treatments

We combined genomic sequence information with available EST data to design primers for the longest possible probes and were able to subclone and synthesize Dig-labeled antisense probes for 73 of the 104 identified genes. 49 of the 73 genes had never been characterized before in *N. vectensis*. In order to analyze their expression pattern and determine their putative implication in the *N. vectensis* endomesoderm GRN, we performed *in situ* hybridization focusing on the late blastula stage (24 hpf) (Figure 4). This embryonic stage is the same stage that was used to perform the initial microarray experiments that lead to the identification of the genes and corresponds to the timing in which the presumptive endomesoderm is specified.

We identified 18 new genes expressed in defined domains within the presumptive endomesoderm (Figure 4A–4R) that were upregulated by treatments described to affect cWnt signaling. Two genes (*Nvhd043* and *Nvngfr-like*) were expressed in the gastrodermis at the late gastrula stage (http://www.kahikai.org/index.php?content=genes) but we were unable to detect differentially localized gene expression for the 29 remaining probes during the first 48 hours of development after fertilization. From the 20 genes that displayed localized expression, eleven were exclusively induced by AZ, five exclusively by LiCl and four by both treatments (Table 2).

Although it was difficult to identify sharp boundaries of expression for a few genes (e.g. *Nv-smad4-like*, *Nv-unc4-like* and *Nv-foxQ1*) at the blastula stage, detailed analysis of animal views of the expression patterns revealed that the newly identified genes could also be characterized as being expressed in one of these two domains (Figure 4A–4R insets) that may constitute distinct synexpression groups [89]. Fourteen genes (*Nvtbx20-like*, *Nvadmp-related*, *Nvvasa-like*, *NvduxABC*, *Nvnk2-like*, *Nvsmad4-like*, *NvelkA-like*, *Nvhd050*, *Nvphtf1-like*, *Nvporcupine-like*, *Nvnk-like 13*, *Nvnfix-like*, *Nvunc4-like and NvfoxQ1* (Figure 4A–4N)) were expressed in the central domain, the transcripts of two genes (*Nvhd147* and *NvbicaudalC-like1* (Figure 4O, 4P) were detected in the central ring surrounding the central region, while *Nvaxin-like* and *Nvnkd1-like* appeared to be expressed in cells spanning both territories (Figure 4Q, 4R).

### Re-analysis of previously published gene expression patterns

In order to establish the ground work for analyzing the gene regulatory network underlying endomesoderm specification/formation that includes the largest possible number of candidate genes, we re-analyzed spatial gene expression with longer probes at 24 hpf (blastula) of 51 formerly published genes (Table S2, highlighted in green). From all re-analyzed genes, we obtained clear expression patterns prior to gastrulation (Figure 5) for 33 genes: the transcription factors *NvotxA*, *NvotxB*, *N-otxC*, *Nvsmad1/5*, *NvsnailA*, *NvsnailB*, *Nvgli*, *Nvgsc*, *NvhlxB9*, *NvashB*, *Nvevx*, *Nvbra*, *NvfoxA*, *NvfoxB*, *Nvtcf*, *Nvlmx*, *Nvlhx1*, the signaling molecules and receptors, *Nvfgf8A*, *Nvfz10*, *Nvbmp2/4*, *Nvwnt3*, *Nvwnt2*, *Nvwnt4*, *Nvwnt8*, *NvwntA*, *Nvstrabismus* the modulators of FGF and BMP signaling, *Nvsprouty*, *Nvtolloid*, *Nvchordin* and putative germ line specific markers *Nvpl10*, *Nvnanos2*, *Nvvasa1 and Nvvasa2*. In addition, the genes *Nvactivin*, *NvmoxD*, *Nvrepo*, *Nvwnt1*, *Nvwnt11*, *and NvWnt16* [80], [81], [90]–[92] show faint expression in the animal hemisphere but require additional analysis to confirm a localized expression at the blastula stage (data not shown).

Systematic analysis of animal views of the obtained expression patterns allowed us to extend the number of genes that belong to the above-mentioned co-expression groups within the animal hemisphere. Eighteen genes *NvotxA*, *NvotxB*, *NvotxC*, *Nvpl10*, *Nvsmad1/5*, *Nvnanos2*, *NvsnailA*, *NvsnailB*, *Nvsprouty*, *Nvvasa1*, *Nvvasa2*, *Nvgli*, *Nvgsc*, *Nvfgf8A*, *Nvfz10*, *Nvtolloid*, *NvhlxB9* and *Nvevx* (Figure 5A–5R) are expressed in the central domain. The transcripts of nine genes *Nvwnt3*, *Nvbmp2/4*, *Nvbra*, *NvfoxA*, *NvfoxB*, *Nvwnt8*, *NvwntA*, *Nvtcf*, and *Nvlmx* (Figure 5S–5Za) are detected in the central ring surrounding the central domain, while *NvashB*, *Nvstrabismus* appeared to be expressed in cells spanning both territories (Figure 5Zb, 5Zc). The genes *Nvwnt4*, *Nvwnt2*, *Nvlhx1 and Nvchordin* are expressed in a third domain defining the animal hemisphere, the external ring (Figure 5Zd–5Zg).

While we confirmed localized expression at the blastula stage for *NvotxB*, *Nvsmad1/5*, *NvsnailA*, *NvsnailB*, *Nvsprouty*, *NvfoxA*, *NvfoxB*, *Nvtcf*, *NvashB* and *Nvlhx1*, (Figure 5B, 5E, 5G, 5H, 5I, 5V, 5W, 5Z, 5Zf) [26], [39], [40], [42], [48], [50], [61], [81], [93]–[95] we also detected an earlier onset of gene expression than previously reported for *NvotxA*, *NvotxC*, *Nvpl10*, *Nvnanos2*, *Nvvasa1*, *Nvvasa2*, *Nvgli*, *Nvgsc*, *Nvfgf8A*, *Nvfz10*, *Nvtolloid*, *NvhlxB9*, *Nvevx*, *Nvwnt3*, *Nvbmp2/4*, *Nvbra*, *Nvwnt8*, *NvwntA*, *Nvlmx*, *Nvwnt4*, *Nvwnt2* and *Nvchordin* (Figure 5A, 5C, 5D, 5F, 5J, 5K, 5L, 5M, 5N, 5O, 5S, 5T, 5U, 5X, 5Y, 5Zb, 5Zc, 5Zc) [40], [56], [61], [80]–[82], [90]–[92], [96]–[100] (Table S2).

**Figure 6:**
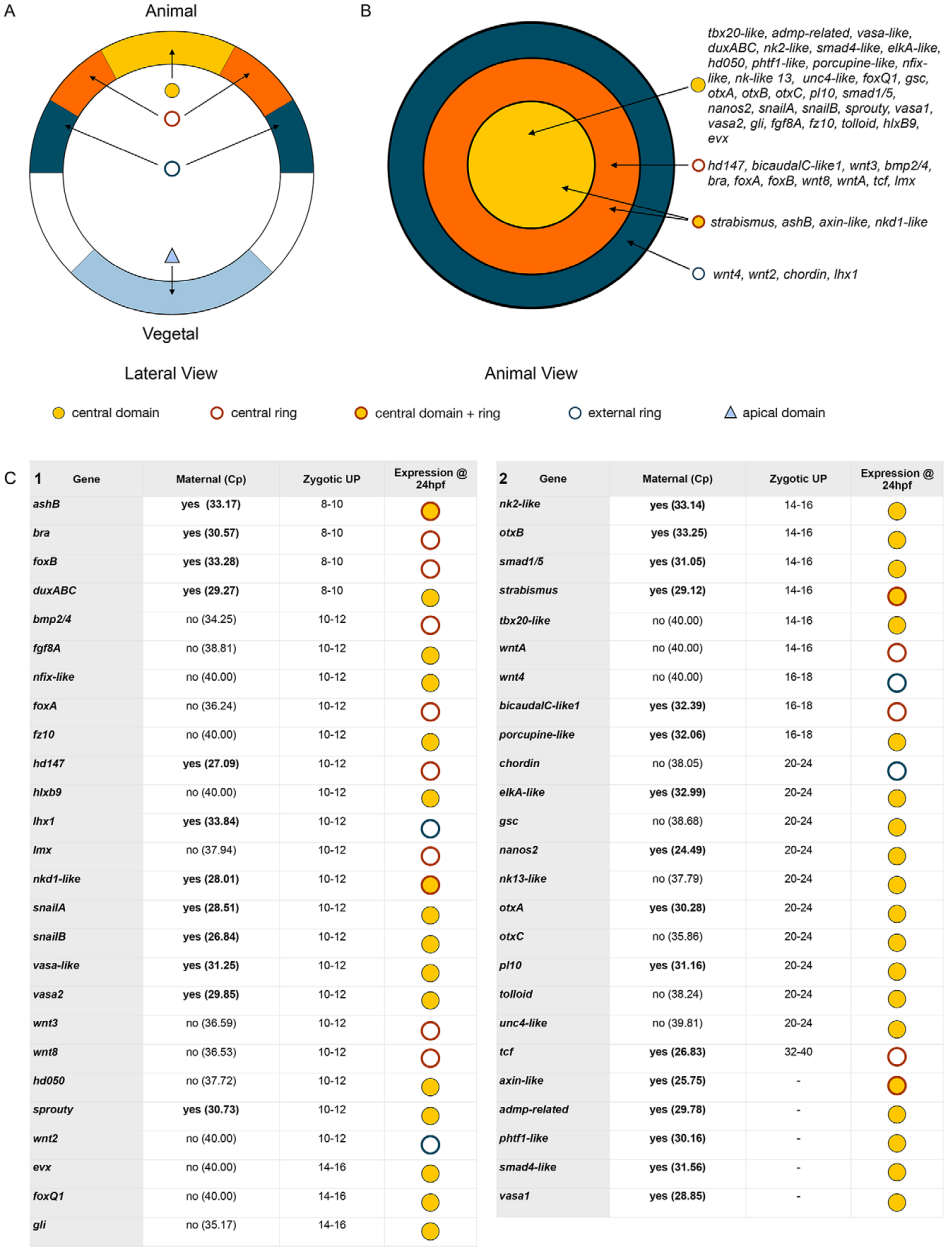
Co-expression domains in the *N. vectensis* blastula and high-density gene expression profiling. (A,B) The animal hemisphere contains at least four domains defined by differential gene expression: the central domain, the central ring, the central domain+ring and the external ring. In the vegetal hemisphere, we identify only one domain, the apical domain. The gene names next to the diagram correspond to the genes expressed in each domain at the blastula stage as examined in this study. (C) Summarized results of the temporal high density profiling (qPCR) used to determine the presence of maternal transcripts and significant zygotic upregulation of a given gene expressed within the animal hemisphere (see Figure S5 for details). Visual keys used to describe the spatial expression domain determined by *in situ* hybridization at 24 hpf same as in A,B. Those genes (n = 19) that were positively affected by Gsk3ß inhibition as determined by our array experiments but for which no localized endomesodermal expression was observed by *in situ* hybridization at 24 hpf are shown in Figure S6.

Taken together, our systematic gene expression analyses of 18 new and 33 previously identified genes (Figure 4, Figure 5) define at least four complementary expression domains (central domain, central ring, central domain+ring, external ring) within the animal hemisphere at the blastula stage (Figure 6A, 6B).

### Fine-scale temporal analysis of endomesodermal genes

Because *in situ* hybridizations are not the most sensitive way to detect the onset of gene expression we used qPCR in order to gain a more precise idea about the temporal expression on cDNA made at embryonic stages sampled every two to four hours, up to 48 hpf. As a frame of reference, embryos at 8 hpf, 18 hpf and 24 hpf contain approximately 430, 2160 or 3480 nuclei respectively (Figure S4). Collected data were analyzed for the presence of maternal transcripts (Cp value>34.00) in unfertilized eggs and, if detectable, for their first zygotic expression inferred from positive changes in transcript levels (Figure 6C, Figure S5). Maternal transcripts were detected for 42.5% (31/73) of the analyzed genes, no significant zygotic upregulation observed for 8.2% (6/73) while only one maternally expressed gene, *Nvtcf*, appears to be zygotically expressed after the onset of gastrulation 32–40 hpf (Figure 6C). The remaining genes (89%, 65/73) are zygotically upregulated between 8 and 24 hpf, *with NvashB*, *Nvbra*, *NvfoxB*, *NvduxABC* (Figure 6C), *Nvhd043*, *Nvhd032 and NvmoxC* (Figure S6A) being the first upregulated genes 8–10 hours post fertilization. Zygotic expression of 29 genes (*Nvbmp2/4*, *Nvfgf8A*, *Nvnfix-like*, *NvfoxA*, *Nvfz10*, *Nvhd050*, *Nvhd147*, *NvhlxB9*, *Nvlhx1*, *Nvlmx*, *Nvnkd1-like*, *NvsnailA*, *NvsnailB*, *Nvvasa-like*, *Nvvasa2*, *Nvwnt2*, *Nvwnt3*, *Nvwnt8*, *Nvsprouty* (Figure 6C), *Nvactivin*, *NvfoxA/B-like*, *Nvhes3*, *Nvtwist*, *Nvwnt1*, *Nvwnt11* and *Nvwnt16* (Figure S6A) are detected only a couple of hours later, 10–12 hpf (Figure 6C, Figure S6A). An additional three waves of zygotic upregulation were observed at 14–16 hpf (*Nvevx*, *Nvfoxq1*, *Nvgli*, *Nvnk2-like*, *NvotxB*, *Nvsmad1/5*, *Nvstrabismus*, *Nvtbx20-like*, *NvwntA* (Figure 6C), *Nvfollistatin-like*, *Nvhd017*, *NvmoxD*, *NvmsxB*, and *Nvrepo* (Figure S6A), 16–18 hpf (*Nvwnt4*, *NvbicaudalC-like1*, *Nvporcupine* (Figure 6C) and *Nvgata* (Figure S6A)), and just prior the onset of gastrulation at 20–24 hpf (*Nvchordin*, *NvelkA-like*, *Nvgsc*, *Nvnanos2*, *Nvnk-like13*, *NvotxA*, *NvotxC*, *Nvpl10*, *Nvtolloid*, *Nvunc4-like* (Figure 6C), *Nvfgf8/17-like* and *Nvtbx15-like* (Figure S6A).

Transcripts of genes zygotically activated during the first 5 waves of expression (8–10, 10–12, 14–16, 16–18 hpf) are localized to one of the four animal hemisphere domains at 24 hpf (Figure 6C, Figure S6). With the exception of *Nvchordin* that is expressed in the external ring, 90% (9/10) of the genes zygotically upregulated at 20–24 hpf are expressed in the central domain, suggesting the beginning of segregation events that define distinct domains within the animal hemisphere at this time of embryonic development in *N. vectensis*.

**Figure 7:**
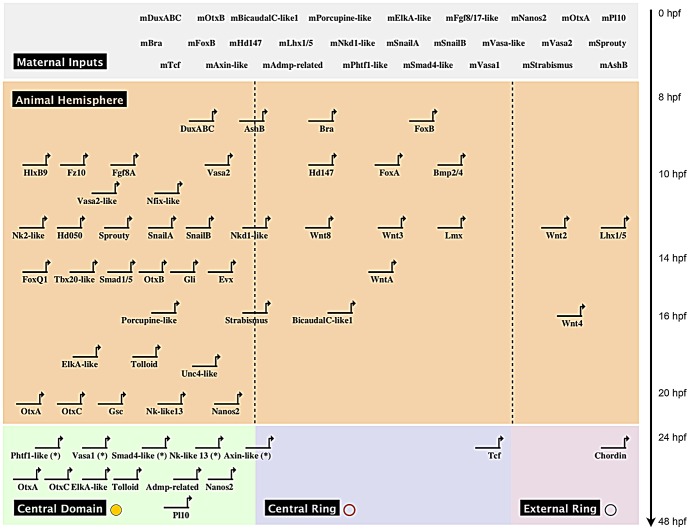
Preliminary spatial and temporal co-expression map. Biotapestry diagram of a preliminary spatial and temporal co-expression map describing the expression data identified in this study. Genes were placed based on their maternal or temporal zygotic appearance as indicated on the y-axis (see arrow on right) and spatial expression domains identified in Figure 6A, 6B as indicated on the x-axis. The dashed lines virtually separate the animal hemisphere prior to the blastula stage into three presumptive domains based on the spatial expression at 24 hpf of the given gene. The * next to gene names indicates that no clear zygotic upregulation was detected prior to the onset of gastrulation (Figure 6) and we therefore placed the genes at 24 hpf.

A spatial and temporal co-expression map (Figure 7) summarizes our expression data analysis (*in situ* hybridization and qPCR) and provides a visual representation of the sequential deployment of the putative members of the cnidarian endomesoderm GRN. The distinction of three co-expression domains within the animal hemisphere has only been determined for the blastula stage at 24 hpf (Figure 4, Figure 5). We assume that genes we analyzed that were detected ubiquitously may also have a defined (not necessarily exclusive) role in the presumptive endomesoderm/animal hemisphere prior to gastrulation. We have organized the genes thought to be involved in endomesoderm formation by their maternal presence and zygotic upregulation in presumptive endomesoderm during the first 48 hours of development and by the co-expression group they belong to at 24 hpf (Figure 6A, 6B).

### Morpholino and dominant-negative based knock-down of NvTcf prevents proper pharynx formation

**Figure 8:**
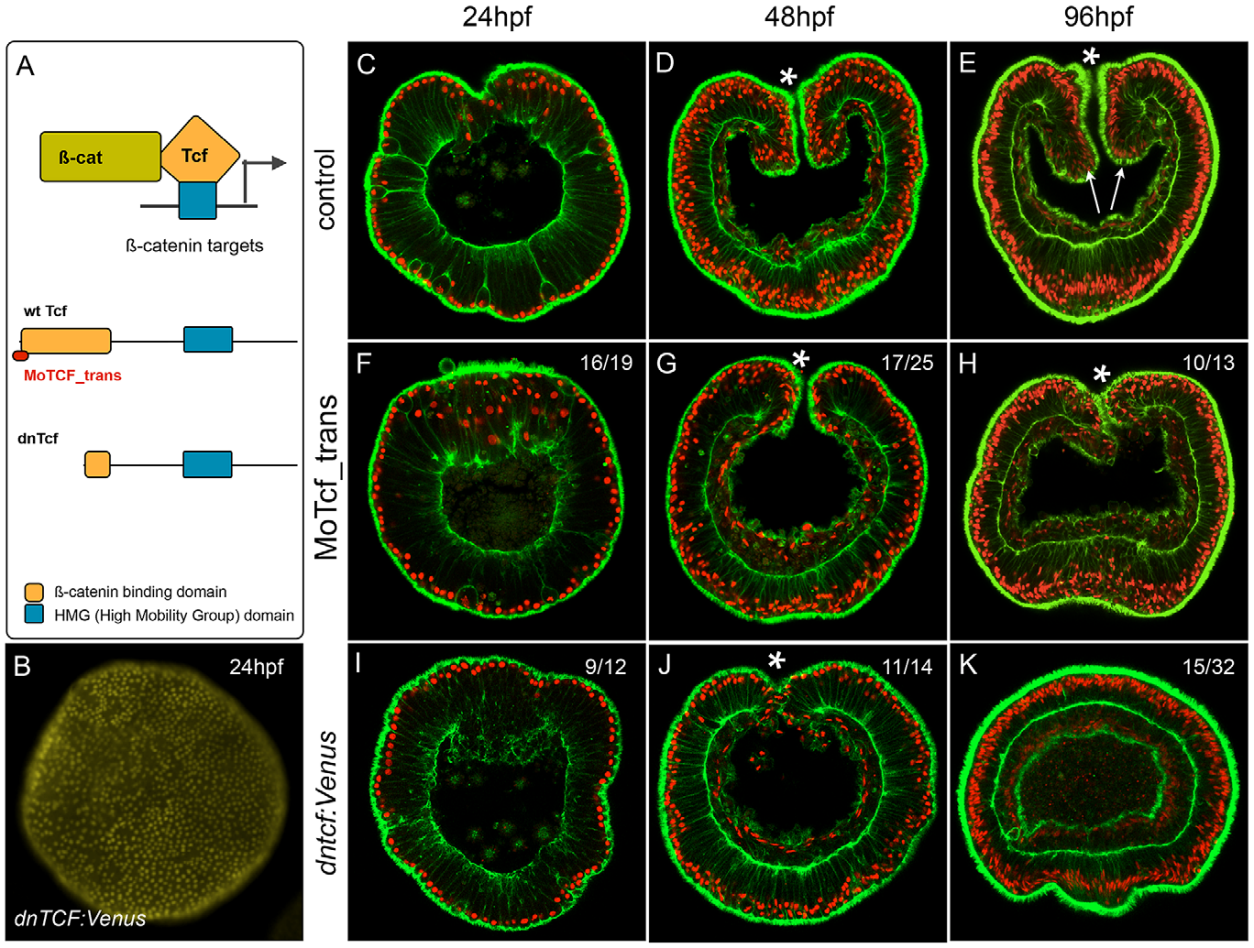
Inhibition of NvTcf prevents pharynx formation. (A) Schematic presentation of the ß-catenin/Tcf interaction for transcriptional activity: the morpholino oligonucleotide MoTcf_trans (red) targeting the translation initiation site of *NvTcf* and the NvdnTcf protein lacking the ß-catenin binding domain which prevents transcriptional activation of NvTcf by ß-catenin. (B) Overexpression of NvdnTcf:Venus is detected in the nuclei of all blastomeres at the blastula stage showing that the nuclear localization of NvdnTcf is not affected by deletion of the ß-catenin binding domain. (C–K) Confocal z-sections using phalloidin (green) to stain f-actin filaments and propidium iodide (red) to visualize the nuclei. (C–E) Control, (F–H) MoTcf_trans injected and (I–K) Nv*dntcf:Venus* injected embryos. (C,F,I) blastula (24 hpf), (D,G,J) late gastrula (48 hpf), (E,H,K) early planula larva (96 hpf). The numbers in the upper right corner indicate the ratio of embryos with the indicated phenotype to the total number of analyzed embryos. The arrows in E indicate the position of the pharynx. All images are lateral views with oral (indicated by *) to the top.

We have shown that treatments designed to ectopically activate the cWnt pathway can be used to identify genes expressed spatially and temporally consistent with involvement in a putative cnidarian endomesodermal GRN. In order to specifically analyze the effect of disrupting canonical Wnt signaling at the phenotypic and transcriptional level in *N. vectensis* and to determine provisional inputs of that pathway into the cnidarian endomesoderm GRN prior to the onset of gastrulation, we injected morpholino antisense oligonucleotides targeting the translation initiation site of the canonical Wnt effector NvTcf (MoTcf_trans) (Figure 8A). While control (Figure 8C–8E) and dextran injected embryos (not shown) gastrulate normally and form distinct pharyngeal structures (arrows in Figure 8E), MoTcf_trans injected embryos (Figure 8F–8H) gastrulate but fail to form a pharynx (Figure 8H). Previous reports using various approaches to inhibit cWnt signaling in *N. vectensis* have shown that the gastrodermis initially forms normally but later loses its epithelial organization [50], [92]. In contrast, in Nv-Tcf morphants, the body wall endomesoderm went ahead and formed a monolayer of epithelial cells (Figure 8H), suggesting only a partial effect of NvTcf knock down.

**Figure 9:**
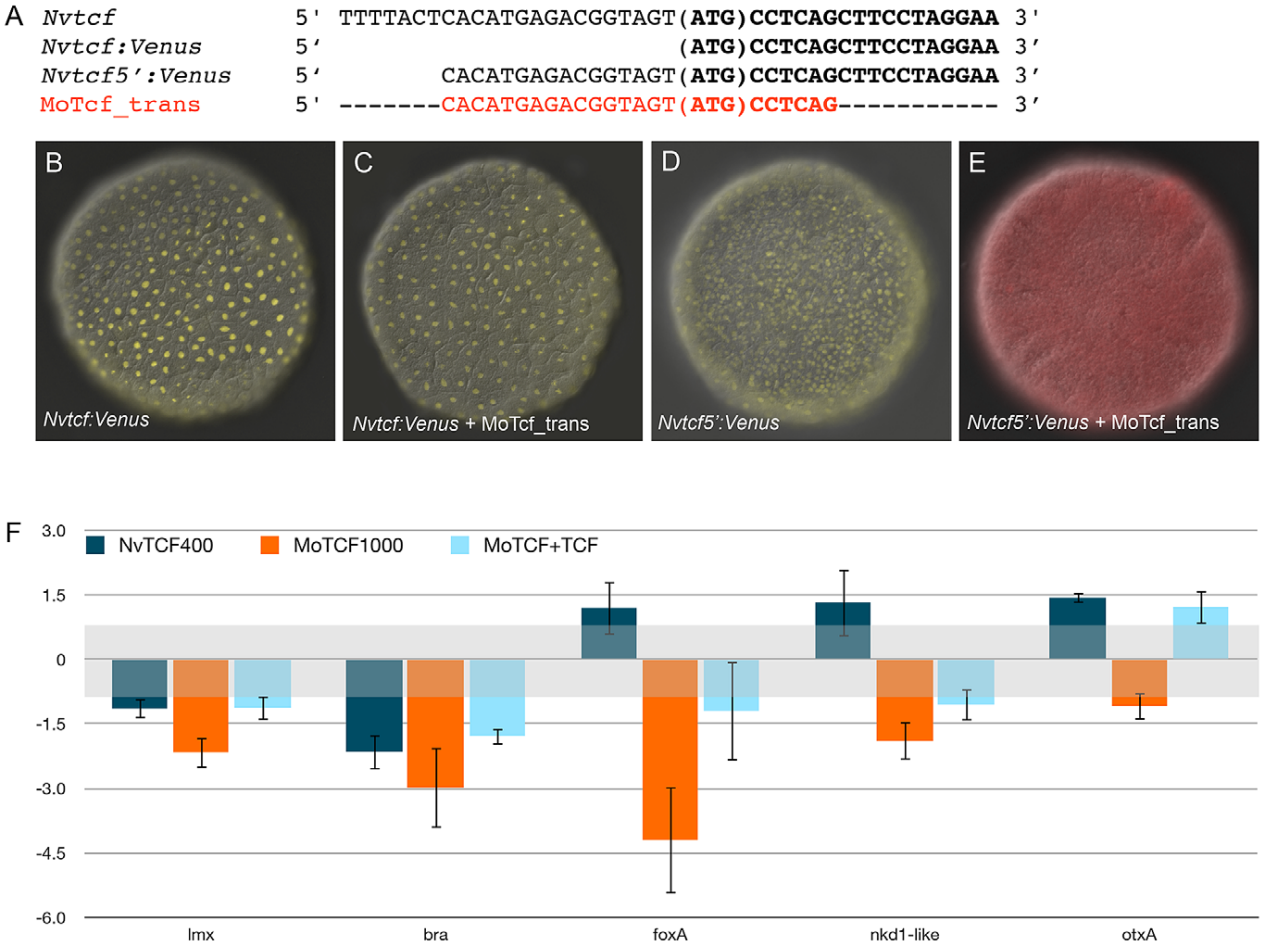
Overexpression of *NvTcf:Venus* can reverse effects of MoTcf. (A) Sequence information for NvTcf (wild-type), NvTcf:Venus (only ORF), NvTcf5′:Venus (containing part of the 5′UTR) and the target sequence for MoTcf_trans. (B) Overexpression of *Nvtcf:Venus* or (D) *Nvtcf5′:Venus* alone or in presence of MoTcf_trans (C,E) showing that MoTcf has no effect on *Nv-tcf:Venus* translation (C), while MoTcf inhibits translation of *Nv-tcf5′:Venus* (E). Note the red color in (E) indicating the dextran that was used for microinjection. The red chanel has been omitted in B,C,D for a better visualization of nuclear Tcf:Venus. (F) Effects on gene expression after injection of *Nvtcf:Venus* (blue), MoTcf_trans (orange) or *Nvtcf:Venus* and MoTcf (light blue). NvTcf:Venus has the capacity to revert the effects of MoTcf_trans supporting the idea that MoTcf specifically targets endogenous *Nvtcf* in the injected embryos.

In order to verify the efficiency of the translational MoTcf_trans that targets a region spanning the 5′ UTR and the translational initiation site of *Nvtcf*, we performed a series of experiments (Figure 9). We made two constructs of NvTcf fused to the fluorescent protein Venus: i) NvTcf:Venus, lacking 15 nucleotides of the morpholino recognition site and ii) Nv-Tcf5′:Venus that contains the entire 5′UTR+ORF region targeted by MoTcf_trans (Figure 9A). When mRNA encoding *Nvtcf:Venus* (400 ng/µl) was injected alone or in presence of MoTcf_trans (1 mM), we observed nuclear localization of NvTcf:Venus in all the cells at the blastula stage (Figure 9B, 9C). In contrast, nuclear localized NvTcf5′:Venus (Figure 9D) was no longer detected when co-injected with MoTcf_trans (Figure 9E). These results show that MoTCF_trans effectively inhibits translation of a synthetic mRNA encoding *Nvtcf* (sequence based on genome prediction corroborated by EST data) and that *Nv-tcf:Venus* mRNA is not recognized by MoTcf_trans making this construct suitable for the following rescue experiments (Figure 9F).

When we injected *Nvtcf:Venus* (400 ng/µl) alone we observed no significant variation in expression of four genes putatively downstream of canonical Wnt signaling (*Nvlmx*, *Nvbra*, *NvfoxA* and *Nvnkd1-like*) by qPCR compared to dextran injected control embryos (Figure 9F). The only exception was *Nvbra*, which was slightly downregulated, reflecting the repressive capacity of Tcf in the absence of nß-catenin [101]. Microinjection of MoTcf_trans (1 mM) causes a downregulation of all four of these genes, while co-injection of *Nvtcf:Venus* together with MoTcf_trans restores similar expression levels compared to the injection of *Nvtcf:Venus* alone (Figure 9F). While *NvotxA* (a gene not affected by ectopic Wnt activation) is slightly upregulated in *Nvtcf_Venus* injections, it remains unaffected following knock-down or rescue conditions (Figure 9F). Taken together, these data support the idea that MoTcf_trans can effectively block translation of *Nvtcf:Venus* and that the observed effects on reduced gene expression in MoTcf_trans injected embryos are primarily caused by the inhibition of NvTcf function (Figure 9F).

*Nvtcf* transcripts are strongly detected in the egg and during early cleavage stages ([56], Figure 6C2) suggesting that the presence of maternally loaded Nv-Tcf protein may circumvent the translational morpholino approach we used to knock-down NvTcf function. In order to interfere with maternally presence of NvTcf, we injected mRNA encoding a dominant negative form of NvTcf fused to Venus (Figure 8A, *Nvdntcf:Venus*) lacking a 92 amino acid region of the N-terminus that contains the ß-catenin binding domain required for proper signal transduction of canonical Wnt signaling [102]. While injection of *Nvdntcf:Venus* into the egg clearly induced nuclear localization of Venus in all cells of the blastula stage (24 hpf, Figure 8B) no effect was observed on early invagination and gastrulation movements (Figure 8I, 8J). However, similar to MoTcf_trans injections, 4 day old *Nvdntcf:Venus* planula larvae (96 hpf) lacked an identified pharynx in over 90% (30/32) of the cases, with no mouth opening observed in appoximately 50% (15/32) of injected embryos (Figure 8K). Intriguingly, in 30% (11/32) of cases we observed various degrees of exogastrulation (Figure S8B, S8C), in addition to the lack of pharynx. When injected at slightly higher concentrations (450 ng/µl) the endomesoderm loses his epithelial organization (Figure S7D), similar to earlier observations of inhibition of cWnt [50], [92] that may eventually lead to apoptosis of the cells [103].

The morpholino (MoTcf) and dominant negative (NvdnTcf:Venus) based approaches we used to interfere with Nv-Tcf function did not perturb gastrulation movements but clearly affected pharynx formation. In *Nvdntcf:Venus* injected embryos we also observed the absence of a mouth opening in addition to a disorganized gastrodermis, supporting the idea that the dominant negative approach interferes with the maternal pool of NvTcf and is thus a more effective strategy to study the role of this gene during early *N. vectensis* development.

### NvTcf knock-down affects expression of genes from all four co-expression groups in the animal hemisphere

#### Molecular readout of NvTcf knock-down by qPCR

**Figure 10:**
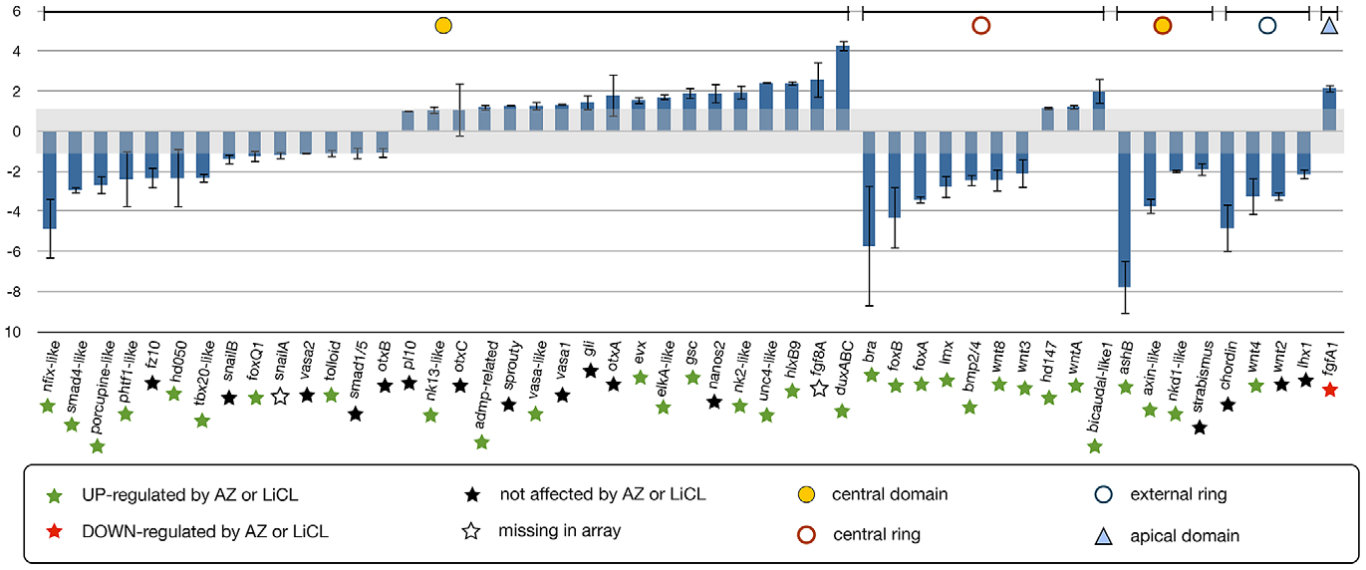
Analysis of NvTcf inhibition by qPCR. Changes in gene expression after NvTcf knock-down (*Nvdntcf:Venus*) compared to control embryos shown by qPCR. Effects of *Nvdntcf:Venus* (blue) overexpression on transcriptional control of 50 potential components of the cnidarian endomesoderm GRN. Changes in gene expression are indicated as relative fold changes compared to dextran injected control embryos (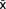 ± sem, n = 3 per gene). The grey bar indicates no significant change in gene expression (−1,1). Below each analyzed gene the star indicates the effects of LiCl or AZ treatments. Gene expression domains at the blastula stage are the same as Figure 6A, 6B. The effects of *Nvdntcf:Venus* and MoTcf_trans injection show an overall similar effect (represented in Figure S8).

In order to determine downstream targets of the cWnt pathway in *N. vectensis*, we disrupted NvTcf function and performed qPCR analysis (Figure 10) on genes expressed in the animal hemisphere (prospective endomesoderm) prior to the onset of gastrulation (24 hpf, Figure 4, Figure 5). Of the 50 endomesodermal genes analyzed, 18 genes (*NvsnailB*, *NvfoxQ1*, *NvsnailA*, *Nvvasa2*, *Nvtolloid*, *Nvsmad1/5*, *NvotxB*, *Nvpl10*, *Nvnk13-like*, *NvotxC*, *Nvadmp-related*, *Nvsprouty*, *Nvvasa-like*, *Nvvasa1*, *Nvgli*, *NvotxA*, *Nvhd147* and *NvwntA*) were unaffected and 22 genes (*Nvnfix-like*, *Nvsmad4-like*, *Nvporcupine-like*, *Nvphtf1-like*, *Nvfz10*, *Nvhd050*, *Nvtbx20-like*, *Nvbra*, *NvfoxB*, *NvfoxA*, *Nvlmx*, *Nvbmp2/4*, *Nvwnt8*, *Nvwnt3*, *NvashB*, *Nvaxin-like*, *Nvnkd1-like*, *Nvstrabsimus*, *Nvchordin*, *Nvwnt4*, *Nvwnt2*, and *Nvlhx1*) were downregulated by *Nvdntcf:Venus* overexpression (Figure 9). Interestingly, ten genes (*Nvevx*, *NvelkA-like*, *Nvgsc*, *Nvnanos2*, *Nvnk2-like*, *Nvunc4-like*, *NvhlxB9*, *Nvfgf8A*, *NvduxABC* and *NvbicaudalC-like1*) were positively regulated by NvdnTcf:Venus (Figure 10) suggesting a repressive function of cWnt on those genes during the first 24 hrs of development.

In order to verify if the slight phenotypic differences observed in MoTcf_trans and *Nvdntcf:Venus* injected embryos (Figure 8) can also be detected by qPCR, we compared the expression of 21 genes after injection of one or the other reagent (Figure S8). All analyzed genes displayed a similar expression regulation after disruption of NvTcf function at 24 hpf. However, these data also confirmed our findings that NvdnTcf:Venus is more efficient in inhibiting NvTcf function than MoTcf_trans injections (i.e. *Nv duxABC*, *Nvbra*, *NvfoxB*, *NvAshB*) probably due to the presence of maternal *Nvtcf* transcripts.

#### Molecular readout of NvTcf knock-down by *in situ* hybridization

**Figure 11:**
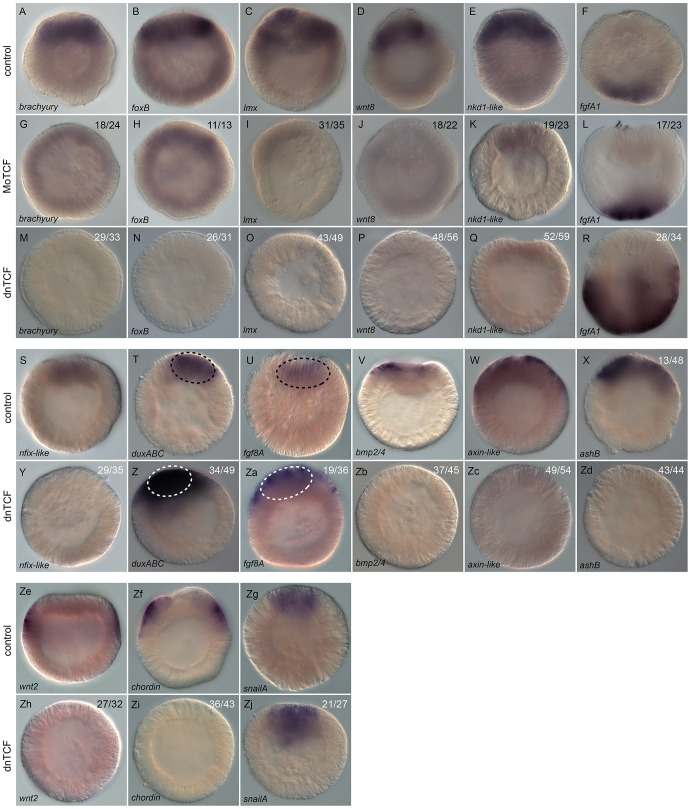
Analysis of Nv-Tcf inhibition by *in situ* hybridization. (A–Zj) Effects on gene expression after MoTcf_trans (G–L) or *Nvdntcf:Venus* (M–R, Y–Zj) injection compared to control embryos (A–F, S–Zg) analyzed by *in situ* hybridization. Antisense probes used as indicated. The black dashed circle in (T,U) indicates the central domain expression of *NvduxABC* and *Nvfgf8A* and the white dashed circle in (Z,Za) the central domain showing extension of its expression domain into the central ring. The numbers in the upper right corner indicates the ratio of embryos with perturbed gene expression to the total number of analyzed embryos. All images are lateral views with the presumptive endomesoderm (animal pole) to the top.

In order to confirm the qPCR results of NvTcf knockdown we also analyzed the effect on NvTcf inhibition on spatial gene expression by *in situ* hybridization on those genes that showed the most dramatic changes (Figure 11). Initially we compared the effectiveness of MoTcf_trans (Figure 11G–11L) and *Nvdntcf:Venus* (Figure 11M–11R) injections on *in situ* expression patterns. In agreement with the qPCR data (Figure S8), the effects appeared more dramatic when using the dominant negative approach. For example, compared to control injected embryos (Figure 11A–11E), *Nvbra* and *Nvwnt8* were strongly downregulated (Figure 11G, 11J), while a faint signal was still detected for *NvfoxB*, *Nvlmx* and *Nvnkd1-like* (Figure 11H, 11I, 11K) in NvTcf morphant embryos. However, in *NvdnTCF* injected embryos expression of all analyzed genes were drastically inhibited (Figure 11M–11P), with the exception of *Nvnkd1-like* that still displayed residual expression (Figure 11Q). Consistent with our qPCR data (Figure S8), ectodermal expression of *NvfgfA1* (Figure 11F) appeared unchanged in MoTCF_trans injected embryos (Figure 11L), while it was enhanced in *Nvdntcf:Venus* injections (Figure 11R).

We further analyzed expression of *Nvnfix-like*, *NvduxABC*, *Nvfgf8A*, *Nvbmp2/4*, *Nvaxin-like*, *NvashB*, *Nvwnt2*, *Nvchordin*, and *NvsnailA* in *NvdnTCF* injected blastula stages (Figure 11Y–Zd, 11Zh–Zj). Expression of genes in the central domain (*Nvnfix-like*, Figure 11S), in the central ring (*Nvbmp2/4*, Figure 11V), genes that span both the central domain and the central ring (*Nvaxin-like* and *NvashB*, Figure 11W, 11X) as well as genes in the external ring (e.g. *Nvwnt2*, *Nvchordin*, Figure 11Ze, 11Zf) were all effectively inhibited (Figure 11Y, 11Zb–11Zd, 11Zh, 11Zi). As predicted by our qPCR data (Figure 10), *NvduxABC* and *Nvfgf8A* were upregulated in NvTcf deficient embryos. However, in control embryos *NvduxABC* and *Nvfgf8A* expression is confined to the central domain (Figure 11T, 11U), while in NvTcf deficient embryos expression of both genes expands to include the central ring (Figure 11Z, 11Za), suggesting that NvTcf represses *NvduxABC* and *Nvfgf8A* expression in the central ring (*Nvtcf* is expressed in this domain at that stage).

We also analyzed *NvsnailA* expression in the central domain that was largely unaffected by NvTCF knock down (Figure 11Zg, 11Zj). This observation was in contrast to previous reports [50], [92] in which *NvsnailA* expression was blocked by cWnt inhibition. This difference might be explained by the timing at which *NvsnailA* expression was analyzed (blastula vs. gastrula), or by the severity of the knockdown. In fact, we only observed loss of endodermal integrity [50], [92] at higher Nv*dntcf:Venus* concentrations (Figure S7).

Taken together, the spatial expression of potential endomesodermal genes confirmed our qPCR data and shows that spatially correct expression of at least fourteen genes (*Nvbra*, *NvfoxB*, *Nvlmx*, *Nvnkd1-like*, *Nvwnt8*, *Nvnfix-like*, *Nvbmp2/4*, *Nvaxin-like*, *NvashB*, *NvfgfA1*, *NvduxABC*, *Nvfgf8A*, *Nvwnt2* and *Nvchordin*) requires functional Tcf signaling in *N. vectensis*. While NvTcf is required for expression in the presumptive endomesoderm of *Nvnfix-like*, *Nvbra*, *NvfoxB*, *Nvlmx*, *Nvnkd1-like*, *Nvwnt8*, *Nvbmp2/4*, *Nvaxin-like* and *NvashB*, it restricts expression of *NvduxABC* and *Nvfgf8A* to the central domain and *NvfgfA1* to the presumptive apical domain (Figure 11). As *NvfgfA1* is expressed in a domain opposite of Wnt/ß-catenin activity, the role of that pathway on patterning the aboral ectoderm may be relayed by a currently unknown signal. We have shown that inhibition of cWnt signaling does not block endomesoderm specification as it only affects pharynx formation and gastrodermal integrity (Figure 8, Figure S7). Furthermore, our results also show that only a subset of the 50 analyzed putative components of the endomesodermal GRN are downregulated prior to gastrulation, suggesting the involvement of additional signaling pathways in the specification of the cnidarian endomesoderm.

## Discussion

In this study we took advantage of the growing number of molecular and functional resources in the cnidarian sea anemone *N. vectensis* to establish the framework for the first provisional GRN underlying endomesoderm (EM) formation in a non-bilaterian metazoan. We used ectopic activation of cWnt signaling (using two different approaches) to identify new putative members of the GRN underlying endomesoderm specification in *N. vectensis*, combined high density temporal gene expression profiling by qPCR as well as detailed spatial expression analysis by *in situ* hybridization to build the framework for the EM GRN. Furthermore, we initiated a functional dissection of potential network components by using antisense oligonucleotide morpholino and mRNA (encoding a dominant negative form of NvTcf) injection to detect downstream targets of Wnt/ß-catenin signaling prior to the onset of gastrulation. The main observations from this study are: i) Gsk3ß inhibition using either AZ or LiCl treatments induces significantly different developmental endomesodermal phenotypes at the morphological and molecular levels, ii) within the animal hemisphere at the blastula stage, *N. vectensis* is already subdivided in at least four co-expression domains prior to the onset of gastrulation, iii) canonical Wnt activation in the animal hemisphere is essential (direct or indirect) for normal expression of some, but not all, genes belonging to all four co-expression groups, iv) cWnt activation appears essential for specifying cell types in the vegetal hemisphere as well as derivatives of the animal hemisphere, and v) that at least two other signaling pathways appear to be involved in particular components of endomesoderm specification.

It is currently too early to make assumptions about the evolutionary changes in network wiring, especially the network circuitry important for particular processes [104] leading to the formation of true mesoderm in bilaterians. Additional gene specific functional and epistasic studies in *N. vectensis* are required to obtain a better understanding of the genetic interactions of endomesodermal genes that will serve as a comparative basis. However, this current study already provides data to point out several conserved features as well as some differences from other endomesodermal GRNs.

### Unexpected differences between AZ and LiCl treatments in *N. vectensis*

The Gsk3ß/APC/Axin protein complex plays a crucial role in regulating the cytoplasmic pool of ß-catenin and inhibition of that complex by its naturally interacting protein, Dsh (disheveled). This complex is also the target of a variety of pharmaceutical drugs causing the activation of canonical Wnt signaling. Historically, lithium chloride (LiCl) was used to inhibit Gsk3 function, mimic Wnt signaling and interfere with sea urchin, zebrafish and *Xenopus* development [105], [106]. While currently more than 30 different pharmalogical Gsk3 inhibitors have been described and characterized biochemically [62] only a handful of reagents (lithium chloride (LiCl), 1-azakenpaullone (AZ), 1-alsterpaulllone (AP) and 6-Bromoindirubin-30-oxime (BIO) are commonly used in developmental and cellular [107] studies. The IC_50_ values (the half maximal (50%) inhibitory concentration (IC) of AZ, AP and BIO are comparable (0.004–0.0018 µM), while LiCl requires higher concentration for effective Gsk3 inhibition (∼2000 µM) [62]. Nonetheless, all four components are broadly used in a variety of animals and generally considered universal canonical Wnt activators [59], [72], [74], [105], [108], [109]. While direct comparisons of two or more Gsk3 inhibitors in a single organism are sparse, recent studies in *Hydractinia* primary polyps (hydrozoan cnidarian) [110], or acoel flatworms [111] have shown that AZ and LiCl or AZ and AP respectively induce similar phenotypes. These results as well as the fact that different Gsk3ß inhibitors are interchangeably used to ectopically activate canonical Wnt signaling in various animals, predict that AZ and LiCl cause comparable developmental perturbations and should affect a largely overlapping pool of downstream targets. Surprisingly, at the molecular level, the genes affected by these treatments in *N. vectensis* are largely non-overlapping and closer analysis of the morphological phenotype revealed clear differences. While AZ causes an exogastrulation (Figure 2L), LiCl treated embryos become elongated and the internal endomesodermal tissue disorganized (Figure 2H). Both treatments enhance *Nv-foxB* expression at the blastula stage at the working concentrations (Figure 1D, 1Q) but only AZ has drastic effects on *Nv-fgfa1* at the vegetal pole (Figure 1J, 1W). A higher concentration of LiCl is needed to visibly reduce *Nv-fgfa1* expression (Figure 1K). Our array data show that only approximately 11% of significantly upregulated genes or 25% of significantly downregulated genes are simultaneously affected by AZ and LiCl treatments (Figure 3A, 3B). One plausible explanation for this observation would be that the concentrations used for the treatments only cause a partial overlap of common targets. However, although only two biological replicates were performed, and the Pearson's correlation factors between biological replicates were low (0.53 and 0.42 for the AZ and LiCl arrays respectively), both our molecular and morphological observations of different phenotypes caused by LiCl or AZ treatment (Figure 1, Figure 2), suggest that these drugs might have radically different modes of action during *N, vectensis* development. A greater understanding of targets of LiCl action might also lend insight into additional inputs of endomesoderm specification acting in parallel to other signaling systems.

A recent study on *N. vectensis* suggests that continuous AP treatments for the first 48 hours after fertilization induces a phenotype that is similar to LiCl treated embryos [59]. While the duration of drug application by the authors was different from the continuous treatments of AZ or LiCl in our study, the described similarities between AP and LiCl add another level of confusion on what pharmaceutical drug to use to mimic ectopic canonical Wnt signaling. Interestingly, overexpression of a constitutively active form of ß-catenin, Xßcat69:GFP, causes exogastrulation (Figure 3F) similar to AZ treatments (Figure 2L). These data suggest that AZ may better mimic ectopic activation of ß-catenin than LiCl (and perhaps AP) in *N. vectensis*. The differences in morphological phenotypes and molecular targets revealed by our array experiments also highlight that these drugs may have additional non-canonical Wnt specific targets in addition to the effect on Gsk3. A broader comparative study that includes a wide range of different Gsk3 inhibitors would be beneficial to better understand which component actually mimics cWnt activation *in vivo*. Because AZ and LiCl treatment generate different phenotypes and molecular responses, it raises concerns about the interpretation of experiments made with pharmacological treatments, and underlines the importance of gene specific knock-down experiments for making concrete statements about gene function.

The observation that some genes upregulated by AZ/LiCl treatments were also upregulated by NvTcf inhibition (and not downregulated as expected, Figure 10, Figure 11Z, 11Za) further illustrates how misleading ectopic activation experiments that are not followed up by gene specific knock-down analysis can be.

For the sake of identifying putative downstream targets of the canonical Wnt pathway that may be part of the cnidarian endomesoderm GRN, we focused this study on genes that are upregulated by treatment of inhibitors of Gsk3ß and therefore could positively respond to canonical Wnt signaling. However, a total of 538 genes were significantly (2-fold or more) downregulated by ectopic activation of cWnt signaling (Figure 3B, data not shown). One gene that was downregulated in the array data obtained from AZ but remains unaffected in LiCl treatments is a gene expressed in the presumptive apical domain (vegetal pole), *NvfgfA1* (Figure 1W, [61]), supporting the different phenotypes and molecular effects observed by these two treatments (Figure 1, Figure 2). A thorough analysis of genes negatively affected by AZ or LiCl treatments will be the focus of a subsequent paper.

### Deployment of components of the cnidarian endomesodermal GRN

A precise understanding of the timing of gene expression and their spatial distribution in the embryo is crucial in order to gain insight into the architecture of developmental GRNs. As our goal was to determine a large framework for future endomesoderm GRN studies in *N. vectensis*, we carefully analyzed spatial and temporal expression of previously published as well as newly identified genes by *in situ* hybridization and high-density qPCR (Figure 4, Figure 5, Figure 6).

### A mid-blastula transition in cnidarian development?

In some bilaterian embryos, the initiation of the bulk of zygotic gene expression is called the MBT (mid-blastula transition, [112]. While the timing of the MBT seems controlled by the ratio of nuclei to cytoplasm [113]–[115], the pre-MBT embryo is defined by synchronous cell divisions [116], heterochromatically repressed genes [117] and the translation of the maternal pool of mRNA [118]. Interestingly, our systematic gene expression profiling analysis shows that in *N. vectensis* more than 40% of the endomesodermal genes analyzed are expressed maternally (Figure 6). In addition, of the 66 genes for which we detected zygotic upregulation, none were activated earlier than 8–10 hours post fertilization. While we could have simply not identified earlier zygotically controlled genes, these observations suggest that *N. vectensis* undergoes an MBT-like event approximately 10 hours post fertilization. Interestingly, the timing correlates with the previously described end of blastula oscillations and the associated shift from synchronous to asynchronous cell divisions in *N. vectensis* [49]. Additional experiments including a careful analysis of the early cleavage pattern and analysis of the heterochromatic state are however required to better understand the initial zygotic transcriptional control of *N. vectensis*.

### Co-expression groups and cell fate

To determine spatial expression patterns and potential clustering of putative endomesodermal co-expression groups we carried out whole mount *in situ* hybridization at the blastula stage. Figure 6 A, 6B summarizes the presence of at least five clear distinct co-expression groups present in the blastula in *N. vectensis*: Four in the animal hemisphere and one at the vegetal pole (the apical domain). In the animal hemisphere 32 genes are expressed in the central domain, 11 genes in the central ring, 4 genes in a territory that covers both the central domain and the central ring vegetal to the central ring, and 4 in an external ring (Figure 6A, 6B). The existence of co-expression groups in the animal hemisphere is not only of interest for establishing the endomesoderm GRN but also for our understanding of the putative “blastoporal organizer” in cnidarians. In fact, a recent work using ectopic grafting experiments has shown the potential of the *N. vectensis* blastoporal lip (a derivate of the central and external rings) to induce a secondary axis suggesting an expression of the same subset of signaling molecules in cnidarian and chordate blastoporal lips as axial “organizers” [119]. While our analysis allowed us to cluster gene expression patterns at the blastula stage to one of the co-expression groups, double *in situ* hybridization experiments are required to better understand the spatial relationship between genes on a cell-by-cell basis.

A previous study from *N. vectensis* has shown by double *in situ* hybridization that the expression domains of the *Nvsnail* (central domain) genes and *NvfoxA* (central ring) at the blastula/early gastrula stages do not overlap and proposed that their boundary can be viewed as the boundary between the endomesoderm and ectoderm [48]. In later stages (gastrula/early planula) *NvsnailA* and *NvsnailB* are expressed in body wall endomesoderm [26], [39] while *NvfoxA* is detected in ectodermal portions of the pharynx and the mesenteries [26], [39]. In order to verify the generality of this observation, we compared genes expressed at blastula stages in either the central domain or the central ring, to their expression at the late gastrula/early planula stage (if data available, Table S3). Of the 32 genes expressed in the central domain (including *NvsnailA*), 12 genes were detected in endomesodermal structures in later stages, 6 genes were expressed in ectoderm related tissue and two genes were associated with endo- as well as ectodermal territories. On the other hand, of the 11 genes expressed in the central ring (including *NvfoxA*) the majority (8/11) are detected in ectodermal structures and 3 in endomesodermal tissue. While clearly not all genes from this analysis follow a similar pattern to *NvsnailA*, *NvsnailB* and *NvfoxA*, it seems that the gastrodermis forms primarily from the central domain and pharyngeal/oral ectoderm from the central and external ring and support the idea that ectodermal versus endomesodermal structures are being specified prior to the onset of gastrulation. However, transcriptional control of gene expression is context dependent and can quickly change during embryonic development. In fact, *NvashB* is expressed in the central domain and central ring at 24 hpf (Figure 5Zb), is not detectable during gastrula stages but is re-expressed in the blastoporal ectoderm in planula stages, suggesting differential transcriptional control during embryogenesis [94]. Therefore using gene expression domains at 24 hpf does not provide a clear answer to the cellular fate of the central domain or ring, or their relationship to an ectodermal-endomesodermal boundary. Labeling of the cells belonging to either of the co-expression groups and following them over time is required to definitively address this question.

### Network architecture

The comparison of gene expression domains in *N. vectensis* also reveals something subtler about regional patterning during early development relative to other systems studied. In echinoderms, the basic principle for the origin of the endomesoderm GRN follows four principal steps. Maternal factors activate (1) endomesoderm specific specification genes in the vegetal hemisphere, which after a signal that induces endo- and mesodermal segregation signal activate (2) two distinct sets of endo- or mesoderm specification genes that in turn inhibit (3) the reciprocal specification genes in a given tissue and activate (4) the germ layer specific differentiation genes [9], [120]. This would suggest that in sea urchins once the mesodermal germ layer is differentiated, its specification genes are either downregulated or maintained at basic levels while differentiation genes are upregulated. At the same time endoderm specification genes have to be strongly downregulated in the mesodermal germ layer so as not to interfere with its own specification program. Therefore, no specification genes are expressed in either one or the other germ layer after the segregation signal. The current version of the echinoderm endomesoderm GRN is in agreement with this idea (http://sugp.caltech.edu/endomes/). Our observations in *N. vectensis* suggest significant differences in the GRN architecture. Not only are endodermal and mesodermal genes expressed in the same gastrodermal precursors (e.g. not repressing each other) (Table S3) but genes of the presumptive endomesoderm (central domain) are later expressed in derivatives of the central ring (ectoderm) and vice versa. These data suggest that in *N. vectensis* the feedback loop mechanisms for segregation and subsequent specification of two distinct germ layers (endo- and mesoderm) are not operating as they are in triploblastic (bilaterian) animals.

### Network kernel

Comparisons of the endomesoderm GRNs from sea urchins and sea stars suggested the existence of a network “kernel”: a conserved GRN subcircuit of five regulatory genes (*blimp1*, *otx*, *bra*, *foxA* and *gataE*) that are tightly linked by positive feedback loops. This kernel is required upstream of initial endomesoderm specification and if expression of any of the genes is perturbed, endomesoderm specification is disrupted [17]. In *N. vectensis*, no *Nvblimp1* orthologue is expressed prior to the end of gastrulation (Ormestad & Martindale, unpublished) and *Nvgata* is not expressed in the animal plate at the blastula stage but only in individual cells of the presumptive ectoderm [26]. The temporal expression of *Nvblimp-like* after the initial specification of endomesoderm and the spatial expression of *Nvgata* suggests, that neither of these two transcriptional regulators are part of a putative ancestral kernel for endomesoderm formation. On the other hand, *Nvotx* (*A,B* and *C*), *Nvbra* and *NvfoxA* are all expressed in time and space suggesting that they may play a crucial role in specifying this germ layer in this cnidarian. Knock-down experiments analyzing the individual roles of these transcription factors in connecting the network and germ layer specification will shed light on the question about the existence of an endomesderm “kernel” that precedes the bilaterian split.

### Role of the canonical Wnt signaling in oral-aboral axis establishment and germ layer specification

In order to functionally analyze the role of canonical Wnt signaling during early *N. vectensis* development, we specifically knocked down NvTcf function using an antisense oligonucleotide morpholino and a dominant negative approach. Overexpression of *NvdnTcf:Venus* shows that while canonical Wnt signaling has no effect on gastrulation movements (Figure 8), it is required for germ layer specification (Figure 10, Figure 11), proper pharynx and mouth formation (Figure 8H, 8K) and maintenance of endomesoderm (Figure 8, Figure S7 [50], [92]). The lack of oral structures (pharynx and mouth) is in agreement with the expression of *Nvtcf* in the pharyngeal and blastoporal endomesoderm in late gastrula/early planula stages [56]. One puzzling observation was the exogastrulation phenotype observed in 30% of *NvdnTCF:Venus* injected planula stages (Figure S7), suggesting that a normal pharynx is required for maintaining the developing endomesoderm inside the planula larvae. However, a properly patterned endomesoderm may also be a pre-requisite for the formation of a normal pharynx. Therefore, additional experiments are required to address the question about the relationships between pharyngeal structures and endomesoderm integrity.

In past studies, the role of cWnt signaling in *N.vectenis* has been analyzed by interfering with the function of the cytoplasmic/membrane-bound members of that pathway Disheveled (dsh) and Axin, as well as the over-expression of constructs designed to inhibit ß-catenin function (ß-catenin:engrailed fusion (Xßcat-Eng) or the cytoplasmic domain of Cadherin) [50], [59], [92]. With the exception of Cadherin (whose specificity to cWnt remains unclear, [92]) that blocks gastrulation movements and gut formation, over-expression of the other constructs has no significant effects on early gastrulation movements but clearly prevents maintenance of the gut epithelium. The *NvdnTcf:Venus* injection phenotypes observed in our study are in line with these results. Currently, we cannot rule out that the knock-down experiment from our study, as well as from previous studies [50], [59], [92] are incomplete which may explain the lack of gastrulation phenotype. *NvdnTcf:Venus* injected embryos show a weak downregulation of *Nvstrabismus* (Figure 10), a gene that has been shown to be required for gastrulation movements in N. vectensis [92]. However, the current data in *N. vectensis* [92] and work in another cnidarian [121], [122] suggests that the PCP/Wnt pathway is involved with the morphological aspects of epithelial folding/invagination in *N. vectensis* and that the cWnt pathway is required for activation of a partial subset of genes involved in endomesoderm specification.

### Earliest inputs of canonical Wnt into the cnidarian endomesodermal gene regulatory network

**Figure 12:**
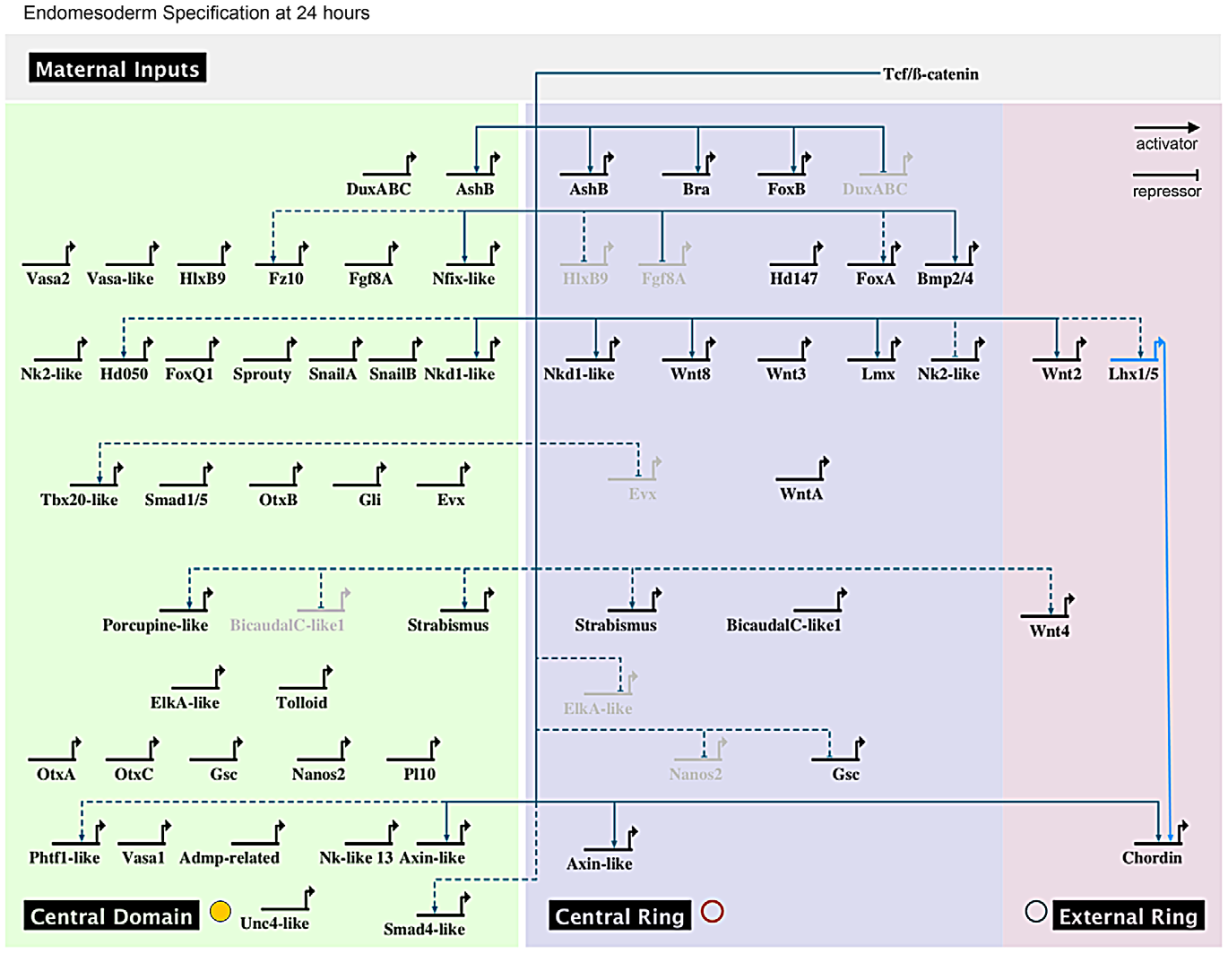
Provisional gene regulatory network orchestrating endomesoderm formation in the cnidarian *N. vectensis*. Biotapestry diagram of the provisional gene regulatory network describing the regulatory interactions of endomesodermal genes identified in this study at 24 hpf. No assumption on whether these interactions are direct or indirect is made. Solid lines indicate functional evidence obtained by qPCR as well as *in situ* hybridization, dashed lines indicate evidence obtained only by qPCR or hypothetical linkages. The colored boxes represent the spatial domains as described in Figure 6A, 6B. The genes that are inactive (repressed in that territory by NvTcf) are represented in light grey. Evidence for NvLhx1/5 controlling *Nv-chordin* expression obtained from Yasuoka et al. 2009.

In this study we combined predicted genome-wide microarray approaches (Figure S1, Figure 3, Table 2, Table S1), with precise temporal and spatial gene expression analysis (Figure 4, Figure 5, Figure 6, Figure 7) as well as *NvTcf* gene specific functional information (Figure 8, Figure 9, Figure 10, Figure 11) to propose the assembly of the framework for the first provisional cnidarian endomesoderm GRN (Figure 12). The current view of endomesoderm specification up to 24 hours post fertilization (Figure 12) allows to clearly distinguish four co-expression domains characterized in this study (Figure 4, Figure 5). No assumptions about direct or indirect interactions are made at this point, and detailed gene specific *cis*-regulatory analyses are needed to address this question in the future.

NvTcf function is required for normal expression of genes belonging to all four co-expression domains of the animal hemisphere (central domain, central ring, central domain+ring and external ring (Figure 6)). An interesting finding is that most of the genes affected by NvTcf inhibition are expressed in the central ring (e.g. *Nvbra*, *NvfoxA*, *Nvbmp2/4* and *Nvwnt8*). This observation is consistent with *NvTc*f expression in that domain at the blastula stage ([56], Figure 5Za) and with the lack of pharynx formation in NvTcf depleted embryos (Figure 8H, 8K). In addition, NvTcf is crucial for regionalizing the animal hemisphere prior to gastrulation. In fact, analysis of the spatial expression of *NvduxABC* and *Nvfgf8A* by *in situ* hybridization shows that central domain expression of both genes is extended to the central ring (Figure 11T, 11U, 11Z, 11Za) suggesting that NvTcf function is required to restrict *NvduxABC* and *Nvfgf8A* expression to the central domain in wild-type embryos.

Endomesoderm GRNs have been proposed for only one protostome (*C.elegans*, [13], [123]) and three deuterostomes (sea urchin, sea star and *Xenopus*, [6], [7], [17], [18], [9], [10], [19]). However, for the sake of simplicity, and because early development between *N. vectensis* and echinoids is in certain aspects comparable [124], we will begin our discussion with echinoderms. However, it is obvious that the GRNs of a broad range of organisms including *Xenopus* and *C.elegans* will need to be included in the future.

### Additional signaling pathways involved in endomesoderm specification

In echinoderms, a maternal canonical Wnt pathway in the vegetal hemisphere plays a crucial role in patterning the animal - vegetal (A/V) axis and is required for endomesoderm specification and gastrulation [55], [58], [125], [126]. In *N. vectensis*, genes from all four animal expression domains are downregulated in NvTcf depleted embryos prior to the onset of gastrulation (Figure 10, Figure 11). However, gastrulation movements and invagination of the endomesodermal germ layer is initiated normally in NvTcf depleted embryos (Figure 8, [50], [59], [92]). One reason for normal endomesoderm formation may be that we did not efficiently block maternal NvTcf proteins or the existence of additional signals that specify the endomesoderm in cnidarians. However multiple functional approaches used to inhibit cWnt all failed to prevent gastrulation (Figure 8, [50], [59], [92]). Interestingly, putative molecules activating other signaling pathways are also expressed in the animal hemisphere prior to gastrulation. *Nvfgf8A* (a putative ligand for Fgf/MAPK signaling) and its putative modulator *Nvsprouty* are both expressed in the central domain ([61], Figure 5I, 5M). *Nvbmp2/4* a putative ligand for Bmp signaling is also expressed in the central ring (Figure 5T) while the potential effector of this pathway *Nvsmad1/5* is expressed in the central domain (Figure 5E).

In echinoderms MAPK and Fgf signaling are required to maintain initial cell-autonomous specification of the skeletogenic mesoderm (primary mesenchyme cells, PMCs), specification of a subset of non-skeletogenic secondary mesenchyme cells (SMCs), PMC ingression and differentiation of the larval skeleton [127]–[130]. In contrast, Bmp2/4 signaling is involved in dorso-ventral (oral-aboral) patterning of all three germ layers after the segregation of the mesoderm from endomesodermal precursors [131]–[134].

The role of Bmps has been recently analyzed in *N. vectensis* and shown a clear implication of NvBmp2/4 in patterning the directive axis (which is perpendicular to the oral/aboral axis) of the endomesoderm and oral ectoderm and patterning and differentiation of the endomesoderm at the late gastrula stages using morpholino approaches [135]. While no delay in gastrulation or morphological signs of a defective endomesoderm was reported from NvBmp2/4 morphants, all endomesodermal markers analyzed in this study were strongly downregulated [135]. This observation is similar to inhibition of cWnt signaling, in that morphogenetic movements of gastrulation and initial gastrodermis formation occurs normally, but endomesodermal markers are no longer detected at the end of gastrulation ([50], [59], [92], this study), suggesting that Bmp2/4 signaling may also be involved in endomesoderm specification prior to gastrulation in *N. vectensis*. As our experiments interfering with the cWnt pathway show, dominant negative approaches might revel additional roles for these other pathways in early endomesodermal patterning.

Unfortunately, little is known about the early role of Fgf/MAPK signaling in the animal hemisphere in cnidarians, it would be important to analyze the role of NvFgf8A signaling on endomesoderm specification in *N. vectensis*. Re-analyzing the role of NvBmp2/4 signaling prior to gastrulation and formation of the directive axis may also reveal whether NvBmp2/4 is involved in endomesoderm specification prior to its role in patterning the directive axis. This would considerably improve our basic understanding of the ancestral relationship between three main signaling pathways (Bmp2/4, Fgf/MAPK, and Wnt/Tcf) and underline their respective inputs into the endomesoderm GRN required to form a functional gut in *N. vectensis*. In the context of our study it appears likely that Bmp2/4 and FGF signaling are likely to be involved in specification of the central domain while Wnt/Tcf is more important for specifying the central ring and its derivatives (e.g. pharynx).

Another very important signaling pathway involved in endoderm and mesoderm segregation from an initial endomesodermal germ layer in echinoderms is the Notch signaling pathway. After initial endomesoderm specification by maternal cWnt, nß-catenin induces the expression of the Notch ligand, Delta, in the presumptive endoderm, which in turn activates the Notch signaling pathways in the neighboring cells (presumptive mesoderm) that actively inhibits cWnt signaling and induces the mesodermal specification program [136]–[138], [8] [16], [139]–[142]. Recently, gene expression of members of the Notch signaling pathway and its role during *N. vectensis* development have been reported [79]. Using pharmaceutical and gene specific approaches to knock-down Notch signaling this study has shown that this pathway is required for proper cnidocyte (cnidarian-specific neural sensory cells) development. While the endomesoderm in Notch inhibited embryos appeared disorganized during later development, expression of two markers (*NvsnailA* and *NvotxA*) was largely unaffected suggesting that initial endomesodermal patterning occurs normally in these animals. This study also suggests that, in contrast to echinoderms, the Notch signaling pathway does not seem to be involved in early germ layer segregation. However, a more detailed analysis of endomesodermal markers prior and during gastrulation after Notch inhibition might be required to fully exclude any important role of that pathway in specifying endomesodermal territories.

To summarize, we have used ectopic activation of canonical Wnt signaling to carry out a genome wide survey of putative members of the cnidarian endomesoderm GRN. In combination with previously described endomesodermal genes we systematically analyzed over 70 genes by *in situ* hybridization and real time qPCR to establish a set of potential components of an extensive gene expression network. Finally we have used functional NvTcf knock-down experiments to assemble the framework for the first provisional inputs into a complex cnidarian gene regulatory network underlying germ layer formation and show that canonical Wnt function is required to regionalize the animal pole into a central domain, central ring and an external ring at the blastula stage and to allow normal pharynx formation of the early planula. The current view of the network suggests that additional signaling pathways (Bmp2/4 and FGF) are tightly interwoven to correctly specify and pattern the endomesoderm of *N. vectensis* prior to the onset of gastrulation.

## Materials and Methods

### Culture and spawning of *N. vectensis*

*N. vectensis* embryos were cultivated at the Kewalo Marine Laboratory/PBRC of the University of Hawaii. Males and females were kept in separate glass bowls (250 ml) in 1/3x seawater (salinity: 12pp) [41]. To keep the animals in a healthy reproductive state, they were kept at 17°C in dark and water was changed weekly. Animals were fed twice a week with oysters or brine shrimps. Manipulating the light cycle induced spawning and oocytes and sperm were collected separately [143]. The gelatinous mass around the eggs was removed with 2–4% L-Cystein in 1/3x seawater before fertilization and then washed 3 times with 1/3x seawater. For a simultaneous development of the embryos, all the oocytes were fertilized in glass dishes at the same time with 0.5 ml of sperm dilution. The fertilized eggs were kept in dark in filtered 1/3 seawater (12pp) at 17°C until the desired stage.

### 1-azakenpaullone and lithium chloride treatments

The canonical Wnt agonist 1-azakenpaullone (AZ, Sigma, #A3734) was dissolved at a stock concentration of 10 mM in DMSO and added at final concentrations as indicted (1–30 µM) in 1/3x-filtered seawater. Lithium chloride (LiCl) was dissolved in H_2_O and added at final concentrations as indicated (1–100 mM) [81]. Embryos were treated with 1-azakenpaullone or lithium chloride directly after fertilization and kept at 17°C. At 12 hours the 1-azakenpaullone and lithium chloride solution were replaced with fresh solutions to maintain activity of the Gsk3ß agonists. The described phenotypes were observed in more than 80% of the analyzed embryos in at least three individual experiments. Treatments were compared to DMSO (for AZ treatments) treated or untreated control embryos. Embryos were fixed for *in situ* hybridization and morphological analysis at indicated stages. mRNA of embryos was extracted at 24 h after fertilization (late blastula) from two distinct biological replicates for microarray analysis.

### RNA extraction, quantitative PCR (qPCR), and microarray analysis

RNA for qPCR and microarray analysis was isolated with TriPure (Roche, # 11667157001) or TRIzol (Invitrogen, #15596-026) according to the manufacturer's instructions and genomic contamination removed using RNase-free DNase (Quiagen, #79254) for 15 minutes at 37°C. The total amount of RNA was quantified with a NanoDrop 2000 spectrophotometer (Thermo Scientific) and the quality analyzed with a Bioanalyzer 2100 (Agilent Technologies Inc.). 1 µg of total RNA was used to generate cDNA with the Advantage RT-PCR kit (Clontech, #639506) for qPCR analysis. For the fine scale temporal analysis (Figure 6, Figure S5, Figure S6) total RNA was extracted from the following stages (in hours post fertilization, hpf): 0,2,4,6, 8,10,12,14,16,18,20,24,28,32,40,48.

qPCR analysis using a LightCycler 480 (Roche) utilizing LightCycler 480 SYBR Green 1 Master mix (Roche, #04887352001) was carried out as described previously [94]. Efficiencies for each gene specific primer pair was determined using a five-fold serial dilution series and only primers with an efficiency ranging from 80% to 115% were used for further analysis (Table S4). The houskeeping genes *Nvactin* and/or *Nvgadph* were used to normalize relative fold changes between control and manipulated embryos and each qPCR analysis was repeated on independent biological replicates. 20 µg of total RNA was sent to NimbleGen, Iceland for further cDNA synthesis, labelling and array hybridization. The 4-plex microarray (72,000 features) is an oligonucleotide-based chip version, custom designed and produced by NimbleGen Systems (Roche). Gene expression levels were normalized in the Nimblescan software according to [144] and [145] and fold-changes calculated by comparing expression values from control and treated embryos.

Array results were screened based on the provided genome annotations assigned to each array spotID. If no clear blast hit or gene information was assigned to the prediction gene model from the Joint Genome Institute, we retrieved the genomic sequences (http://genome.jgi-psf.org/Nemve1/Nemve1.home.html) for the given gene and performed manually Blast (blastx) searches [146] against the NCBI database to determine the nature of the predicted gene product. All sequences from genes of interest have been used for Blast analysis to confirm their nature and to determine previously published genes.

### Nomenclature

To distinguish between previously published genes, and newly identified putative TFs and signaling molecules, we used the best Blast Hit identification, followed by “- like” to designate the newly identified gene sequences. In order to verify the potential accuracy of the “best blast hit” naming system, we used published phylogenetic reconstruction techniques to confirm the orthologies of *Nv-admp-related*, *Nvfgf20-like*, *Nvfgf20-like* as well as forkhead transcription factors (see Table 2 for references). Thus, while “Blast hit” approaches can be used to provide a general idea of the protein family, a detailed phylogenetic analysis is required to better resolve these gene orthologies, especially when paralogy issues or when multiple gene predictions are present for one gene family.

### cDNA construction, mRNA synthesis, NvTcf morpholino design, and microinjection

The constructs pC2+Nvßcat:GFP and pCs2+Xßcat69:GFP have been described previously [59], [71]. cDNA constructs encoding the wild type ORF (NvTcf), the wild type ORF including 16 nucleotides of the 5′UTR (NvTCF5′) and a dominant negative form (NvdnTcf) lacking 276 nucleotides of the 5′ coding sequence of NvTcf, were generated by PCR. The forward primers used were:

NvTcf_FWD (5′ CACC**ATG**CCTCAGCTTCCTAGGAATTCC 3′)

NvTcf5′_FWD (5′ CACCACATGAGACGGTAGT**ATG**CCTCAG 3′)

NvdnTcf_FWD (5′ CACC**ATG**AACCAGCATGGTAGTGACAGTAAAC 3′)

The reverse primer (5′ GTGTCTGATGTTACTGGATTACTTG 3′) used was lacking the stop codon for fusion with a C-terminal Venus fluorescent tag.

NvTcf cDNA constructs were cloned into pENTR dTOPO vectors (Invitrogen) and subsequently recombined into a C-terminal Venus containing pDEST expression vector [147]. pDest expression vectors were linearized with the restriction enzyme ACC651 and transcribed using the Ambion mMessage mMachine T3 kit (Ambion, AM1348). pCs2+ expression vectors were linearized with the restriction enzyme Not1 and transcribed using the Ambion mMessage mMachine SP6 kit (Ambion, AM1340M). Synthetic mRNA was purified using Megaclear columns (Ambion, AM1908) followed by one phenol-chloroform extraction and isopropanol precipitation. *Nvßcat:GFP*, *Xßcat69:GFP*, *NvTCF:Venus*, *NvTCF5′:Venus* and *NvdnTCF:Venus* mRNAs were injected in zygotes at final concentrations of 0.3–0.5 µg/µl.

A morpholino antisense oligonucleotide (Gene Tools) was designed to target a region spanning the 5′UTR and tranlsation inition site of Nv-Tcf (MoTcf_trans: 5′ CTG AGG CAT ACT ACC GTC TCA TGT G 3′, Figure S7). The morpholino was used at 1 mM without noticeable toxicity. Absence of gene expression perturbation after injection of a control morpholino (5′ AGAGGAAGAATAACATACCCTGTCC 3′) at 1 mM has been reported previously [94]. All injections were compared to either rhodamine dextran injected or uninjected control embryos. Microinjections were performed using a PLI-90 Pico-Injector (Harvard Appartus). All embryos developed in 1/3x filtered-seawater at 17°C.

### *In situ* hybridization, actin, and nuclear staining

Previously described gene sequences were used to sub-clone into pGemT (Promega, #A3600) from mixed stage cDNA. All other sequences used in this study were isolated in the course of a microarray analysis. Genome predictions as well as EST sequence information were combined to design primers (Table S5) that allow the amplification and cloning of genes between 05.kb and 2 kb as described above. Accession numbers for all analyzed genes in this study can be found in Table 2.

Embryo fixation, probe synthesis and *in situ* hybridization were performed as previously described [26], [148]. 0.5 kb–2 kb digoxigenin-labelled (Roche, #11573152910) riboprobes were synthesized using the MegaScript Transcription Kit (Ambion). Hybridization of riboprobes (1 ng/µl) was carried out at 62°C in 50% formamide hybe buffer and visualization of the labeled probe was performed using NBT/BCIP as substrate for the alkaline phosphatase-conjugated anti-DIG antibody (Roche, #11093274910). To analyze embryonic and larval morphology, we used Biodipy FL Phallacidin (Molecular Probes/Invitrogen, #B607) and propidium iodide (Sigma, #81845) to stain f-actin and the cell nuclei respectively as described previously [48].

*in situ* hybridization images were taken on a Zeiss AxioScop 2 mounted with with an Axiocam camera triggered by Axiovision software (Carl Zeiss). All expression patterns described here have been submitted to Kahi Kai, a comparative invertebrate gene expression database [149] hosted at http://www.kahikai.org/index.php? content = genes. Scoring of treatment, overexpression and morphant phenotypes was performed on a Zeiss Z-1 Axio imager microscope and confocal imaging was conducted on a Zeiss LSM710 microscope running the LSM ZEN software (Carl Zeiss). Fluorescent images were false-colored, the fluorescent channels merged using ImageJ (http://rsbweb.nih.gov/ij/) and cropped to final size in Photoshop Cs4 (Adobe Inc.).

## Supporting Information

Figure S1 Workflow diagram of the present study. Diagram illustrating the general workflow of this study with reference to the relevant figures.
(PDF)

Figure S2 LiCl and AZ treatments expand nuclear localization of ß-catenin. *Nv-ßcatenin:GFP* or *Xßcat69:GFP* (stabilized form of ß-catenin) mRNA (green, upper row) was co-injected with rhodamine dextran (red, middle row) and then treated with the indicated Gsk3ß inhibitor. The merged images in the bottom row correspond to the images shown in Figure 2A, 2E, 2I; Figure 3C.
(PDF)

Figure S3 AZ treatment causes exogastrulation. Ectopic activation of canonical Wnt after AZ treatments induces exogastrulation four days after fertilization. (A–E) Control, (F–J) AZ treated embryos. Confocal z-sections using phalloidin (green) to stain f-actin filaments and propidium iodide (red) to visualize the nuclei. Stages as indicated in top of the panel. All images are lateral views with oral (indicated by *) to the left.
(PDF)

Figure S4 Number of nuclei that compose early *N. vectensis* embryos. General morphology (confocal z-stacks, see legend Figure 2) and renderings that show the number of nuclei that compose an embryo 8 hrs, 18 hrs or 24 hrs post fertilization (n = 8 per stage). The nuclei were counted using the Imaris software (Bitplane, AG) setting the semi-automatic detection diameter (spot-mode) to 4 µm.
(PDF)

Figure S5 Gene expression analyzed by qPCR (endomesodermal genes). High-density gene expression profiles represented by charts for all genes expressed in the animal hemisphere at the blastula stage (24 hpf) analyzed in this study. Y-axis indicates the relative fold change compared to unfertilized eggs. X-axis indicates developmental time in hours post fertilization. Gene names as indicated in the top left corner and the Cp value in unfertilized eggs is indicated in the top right corner of each panel that was used to determine the presence of maternal transcripts in Figure 7 (Cp>34.00). Cp corresponds to the crossing point (also known as Ct (cycle threshold) value).
(PDF)

Figure S6 Gene expression analyzed by qPCR (additional genes). (A) Summary and (B) charts of high-density gene expression profiles for all genes not expressed (or undetected) in the animal hemisphere at the blastula stage (24 hpf) analyzed in this study. Y-axis indicates the relative fold change compared to unfertilized eggs. X-axis indicates developmental time in hours post fertilization. Gene names as indicated in the top left corner and the Cp value in unfertilized eggs is indicated in the top right corner of each panel that was used to determine the presence of maternal transcripts in Figure S6A (Cp>34.00). Cp corresponds to the crossing point (also known as Ct (cycle threshold) value).
(PDF)

Figure S7 Effects of *Nv-dntcf:Venus* overexpression on *N. vectensis* development. Alternative phenotypes observed after *Nv-dntCF:Venus* injection (B,C) at 300 ng/µl or at a higher concentration, (D, 400 ng/µl) compared to (A) control embryos. Confocal z-sections using phalloidin (green) to stain f-actin filaments and propidium iodide (red) to visualize the nuclei. (A–D) late gastrula stages. All images are lateral views with oral pole (indicated by *) to the top.
(PDF)

Figure S8 Comparative analysis of the molecular effects of MoTcf_trans and Nv-dnTcf:Venus by qPCR. Comparison of the effects on transcript levels after *Nv-dntcf:Venus* (blue) or MoTcf_trans (orange) injection showing an overall similar effect.
(PDF)

Table S1 Genes with at least a 2-fold upregulated after AZ or LiCl treatments based on our array analysis. This Excel file contains two sheets (one for AZ and the other LiCl) identified by tabs at the bottom of the file.
(XLS)

Table S2 List of previously published genes showing gene expression in animal hemisphere related domains.
(XLS)

Table S3 Comparison of expression domains of a given gene at the blastula and the late gastrula/early planula stages. The colors indicate the expression domains at the late gastrula/early planula stages to facilitate comparisons with expression at the blastula stage (24 hpf): yellow (endoderm), blue (ectoderm) and grey (endoderm+ectoderm).
(XLS)

Table S4 Primer pairs used in this study for qPCR analysis.
(XLS)

Table S5 Primer pairs used in this study for gene cloning.
(XLS)
