# Supplementary figures and images for: A Framework for the Establishment of a Cnidarian Gene Regulatory Network for “Endomesoderm” Specification: The Inputs of ß-Catenin/TCF Signaling
Source: PLoS Genet. 2012 Dec 27;8(12):e1003164. doi: 10.1371/journal.pgen.1003164 (PMC3531958; doi:10.1371/journal.pgen.1003164)

Supplementary Figure 1

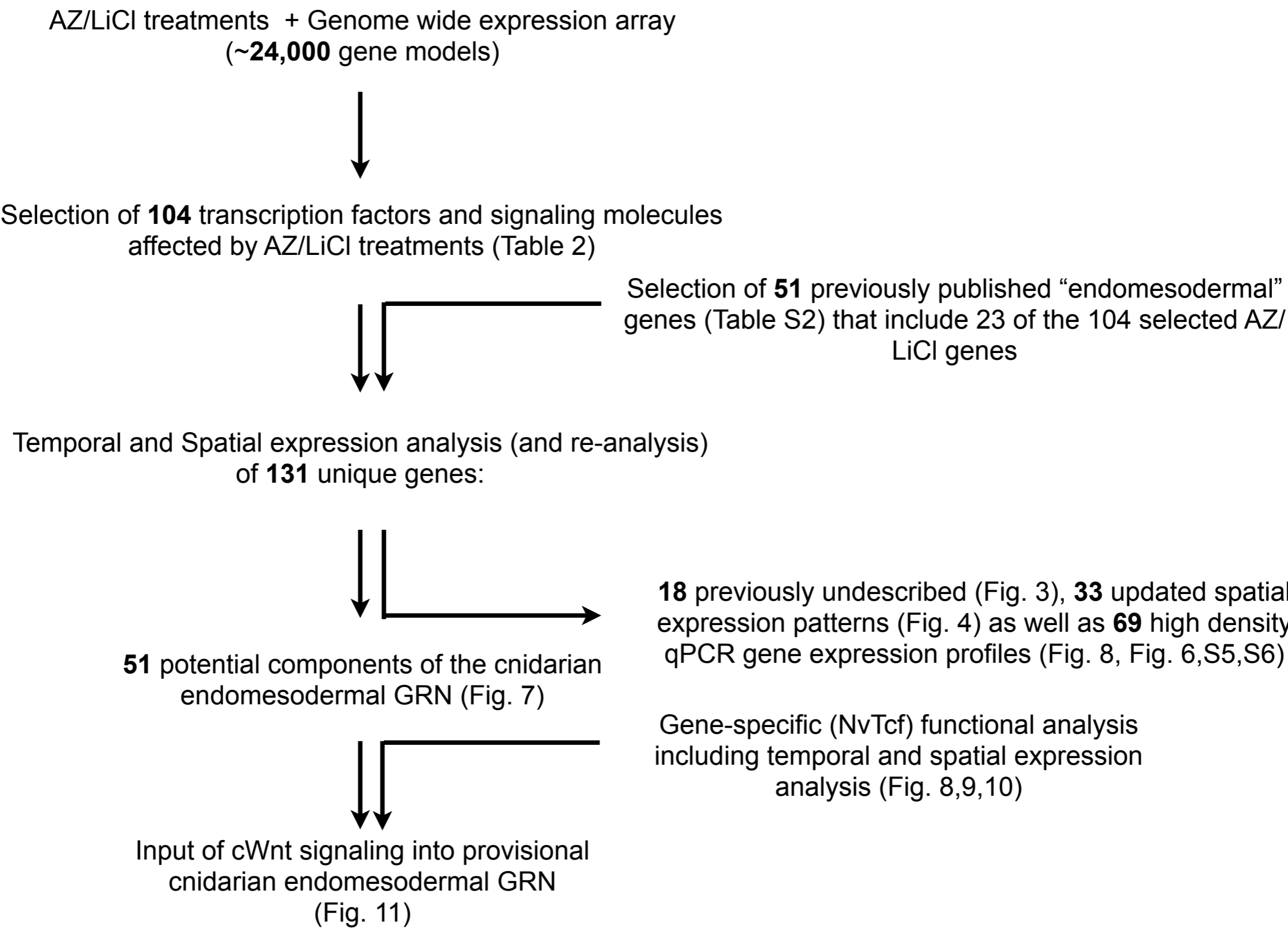

Supplement: Figure S1 — Workflow diagram of the present study. Diagram illustrating the general workflow of this study with reference to the relevant figures. (PDF) [file pgen.1003164.s001.pdf]

Supplementary Figure 2

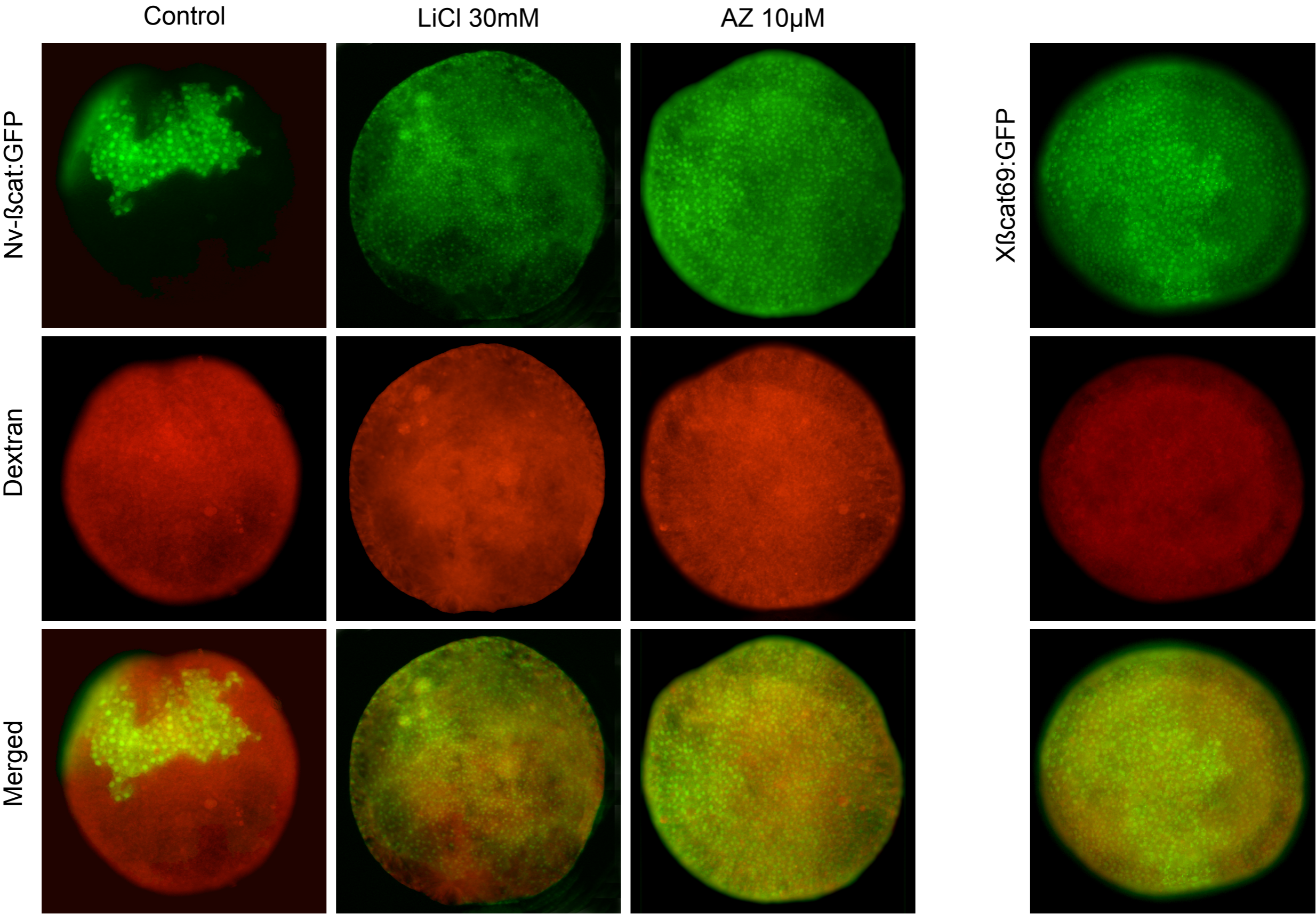

Supplement: Figure S2 — LiCl and AZ treatments expand nuclear localization of ß-catenin. Nv-ßcatenin:GFP or Xßcat69:GFP (stabilized form of ß-catenin) mRNA (green, upper row) was co-injected with rhodamine dextran (red, middle row) and then treated with the indicated Gsk3ß inhibitor. The merged images in the bottom row correspond to the images shown in Figure 2A, 2E, 2I; Figure 3C. (PDF) [file pgen.1003164.s002.pdf]

Supplementary Figure 3

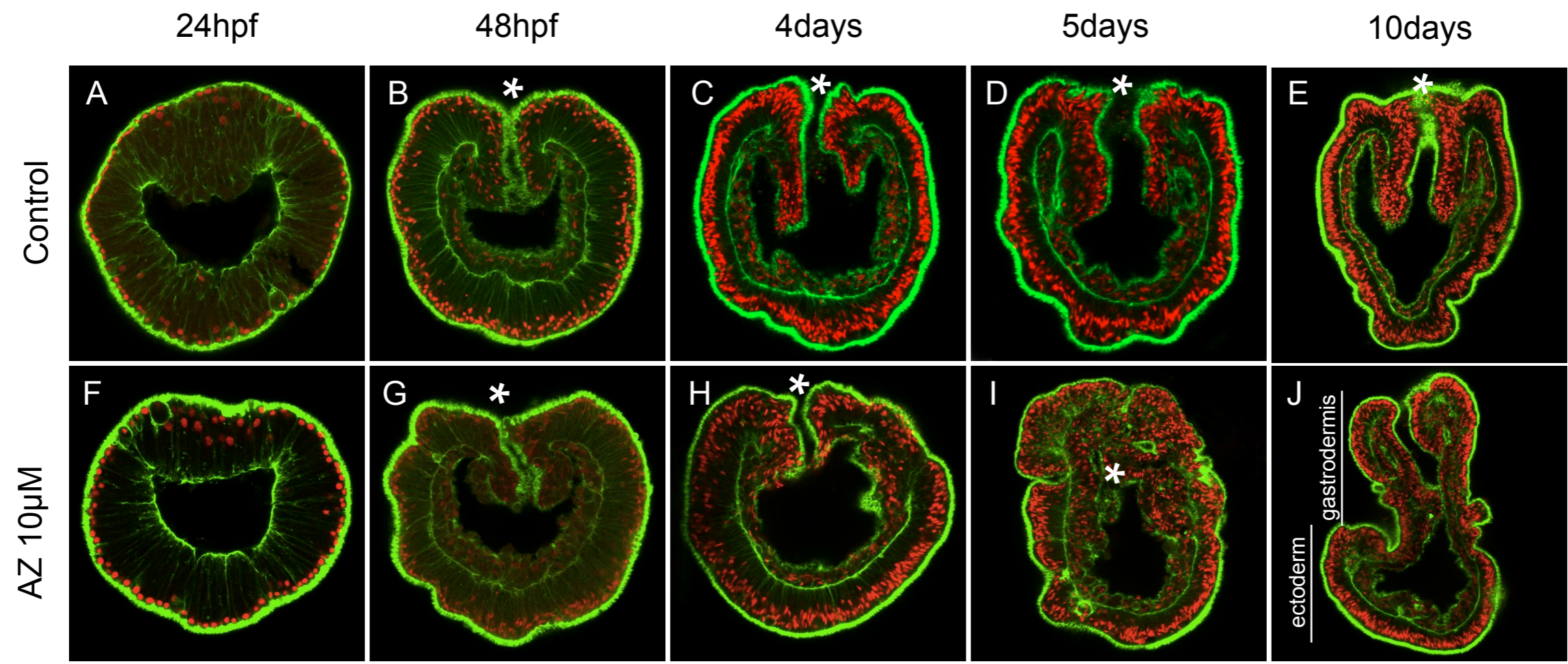

Supplement: Figure S3 — AZ treatment causes exogastrulation. Ectopic activation of canonical Wnt after AZ treatments induces exogastrulation four days after fertilization. (A–E) Control, (F–J) AZ treated embryos. Confocal z-sections using phalloidin (green) to stain f-actin filaments and propidium iodide (red) to visualize the nuclei. Stages as indicated in top of the panel. All images are lateral views with oral (indicated by *) to the left. (PDF) [file pgen.1003164.s003.pdf]

Supplementary Figure 4

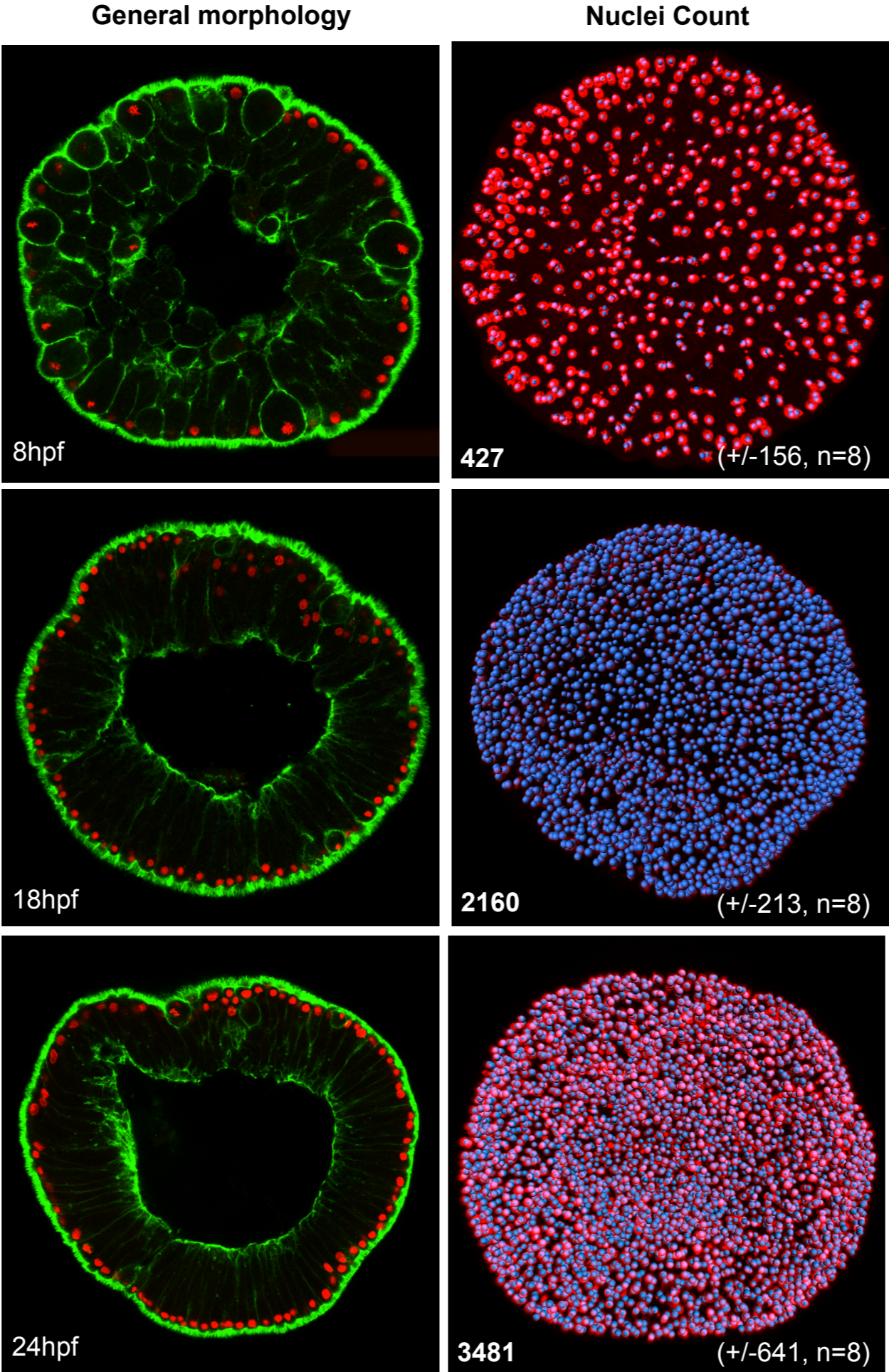

Supplement: Figure S4 — Number of nuclei that compose early N. vectensis embryos. General morphology (confocal z-stacks, see legend Figure 2) and renderings that show the number of nuclei that compose an embryo 8 hrs, 18 hrs or 24 hrs post fertilization (n = 8 per stage). The nuclei were counted using the Imaris software (Bitplane, AG) setting the semi-automatic detection diameter (spot-mode) to 4 µm. (PDF) [file pgen.1003164.s004.pdf]

Supplementary Figure 7

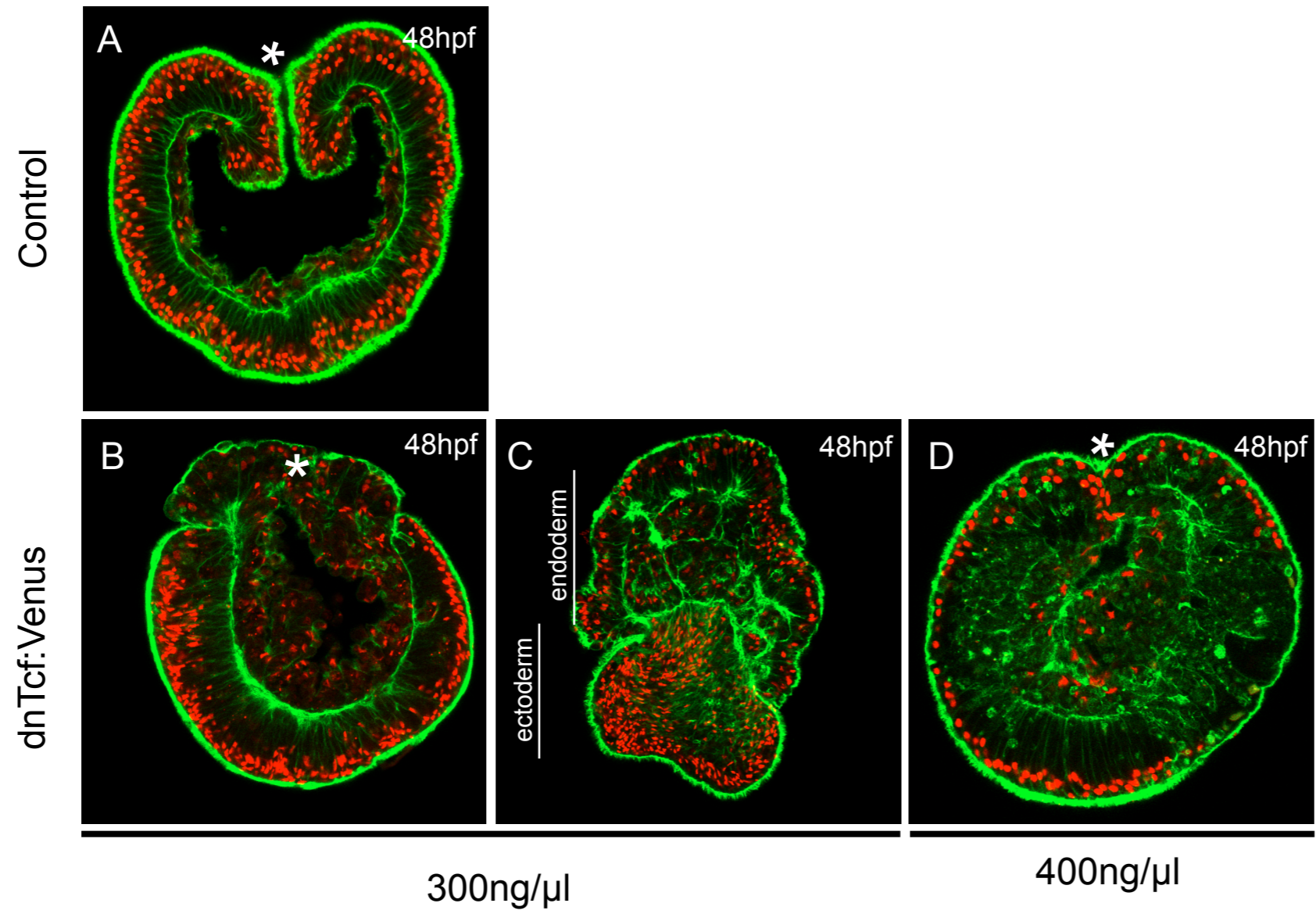

Supplement: Figure S7 — Effects of Nv-dntcf:Venus overexpression on N. vectensis development. Alternative phenotypes observed after Nv-dntCF:Venus injection (B,C) at 300 ng/µl or at a higher concentration, (D, 400 ng/µl) compared to (A) control embryos. Confocal z-sections using phalloidin (green) to stain f-actin filaments and propidium iodide (red) to visualize the nuclei. (A–D) late gastrula stages. All images are lateral views with oral pole (indicated by *) to the top. (PDF) [file pgen.1003164.s007.pdf]
