# Supplementary material for: A Framework for the Establishment of a Cnidarian Gene Regulatory Network for “Endomesoderm” Specification: The Inputs of ß-Catenin/TCF Signaling
Source: PLoS Genet. 2012 Dec 27;8(12):e1003164. doi: 10.1371/journal.pgen.1003164 (PMC3531958; doi:10.1371/journal.pgen.1003164)
Supplement: Figure S5 — Gene expression analyzed by qPCR (endomesodermal genes). High-density gene expression profiles represented by charts for all genes expressed in the animal hemisphere at the blastula stage (24 hpf) analyzed in this study. Y-axis indicates the relative fold change compared to unfertilized eggs. X-axis indicates developmental time in hours post fertilization. Gene names as indicated in the top left corner and the Cp value in unfertilized eggs is indicated in the top right corner of each panel that was used to determine the presence of maternal transcripts in Figure 7 (Cp>34.00). Cp corresponds to the crossing point (also known as Ct (cycle threshold) value). (PDF) [file pgen.1003164.s005.pdf]

Supplementary Figure 5

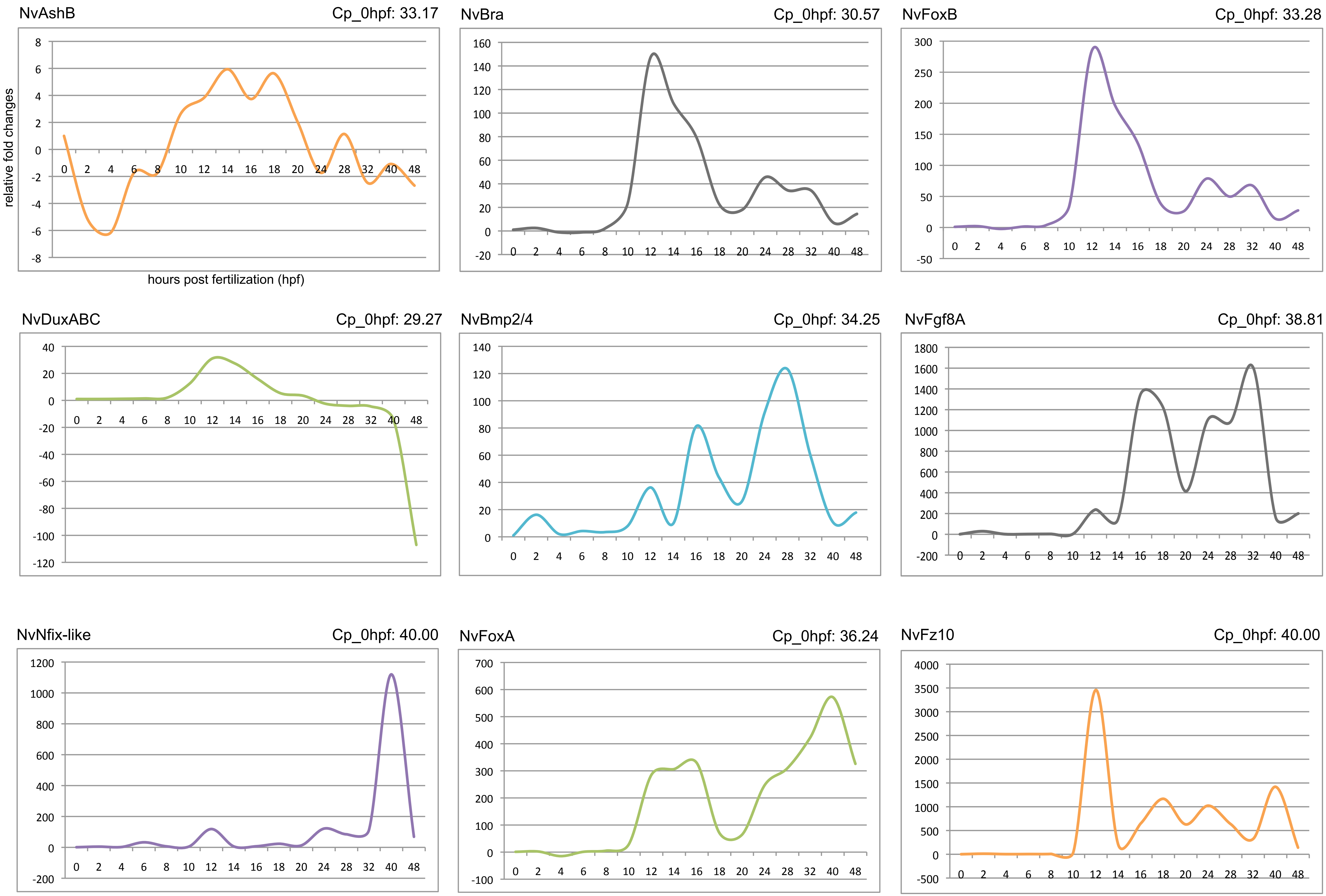

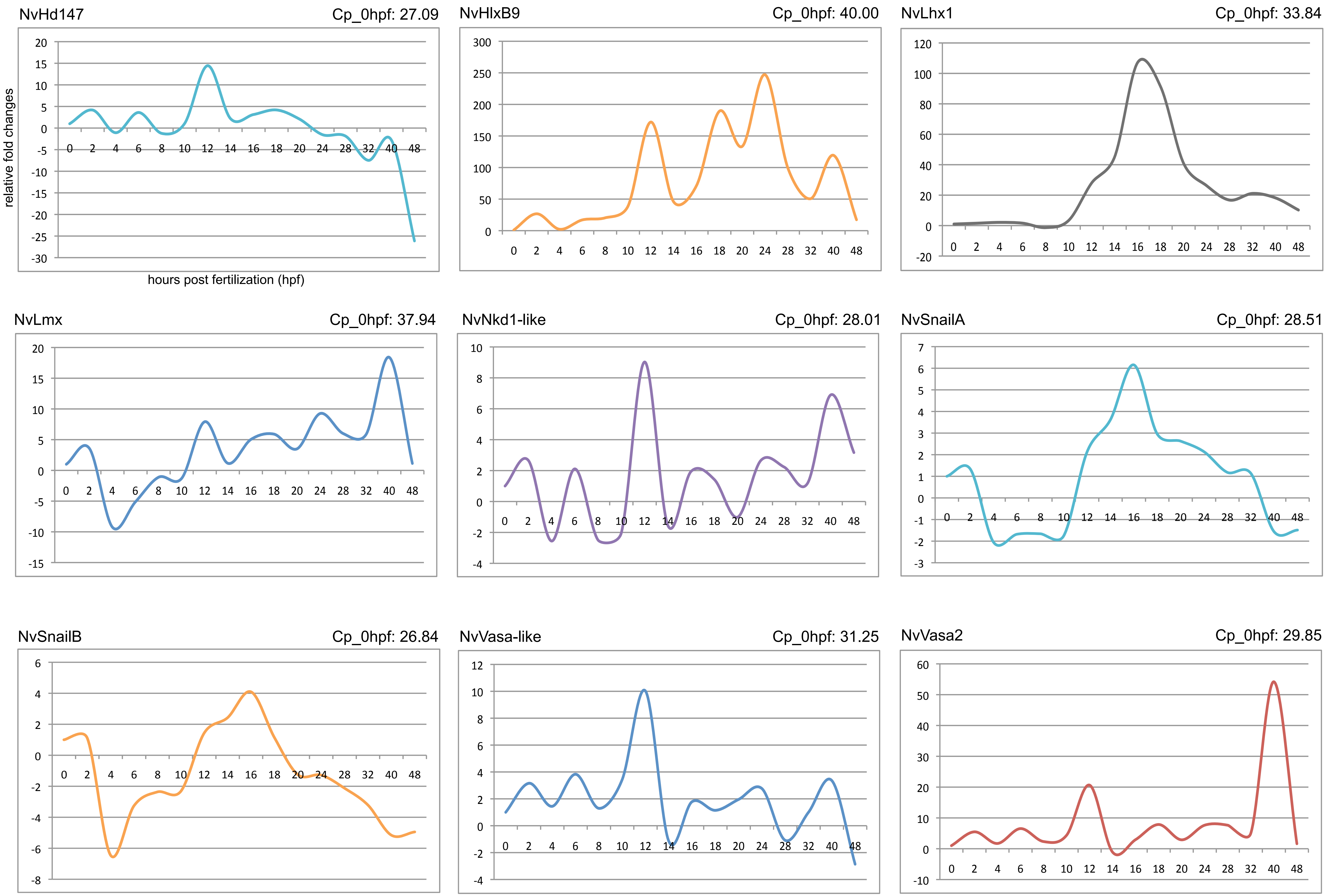
NvHd147
Cp_0hpf: 27.09
relative fold changes
hours post fertilization (hpf)
NvHlxB9
Cp_0hpf: 40.00
NvLhx1
Cp_0hpf: 33.84
NvLmx
Cp_0hpf: 37.94
NvNkd1-like
Cp_0hpf: 28.01
NvSnailA
Cp_0hpf: 28.51
NvSnailB
Cp_0hpf: 26.84
NvVasa-like
Cp_0hpf: 31.25
NvVasa2
Cp_0hpf: 29.85
0 2 4 6 8 10 12 14 16 18 20 24 28 32 40 48

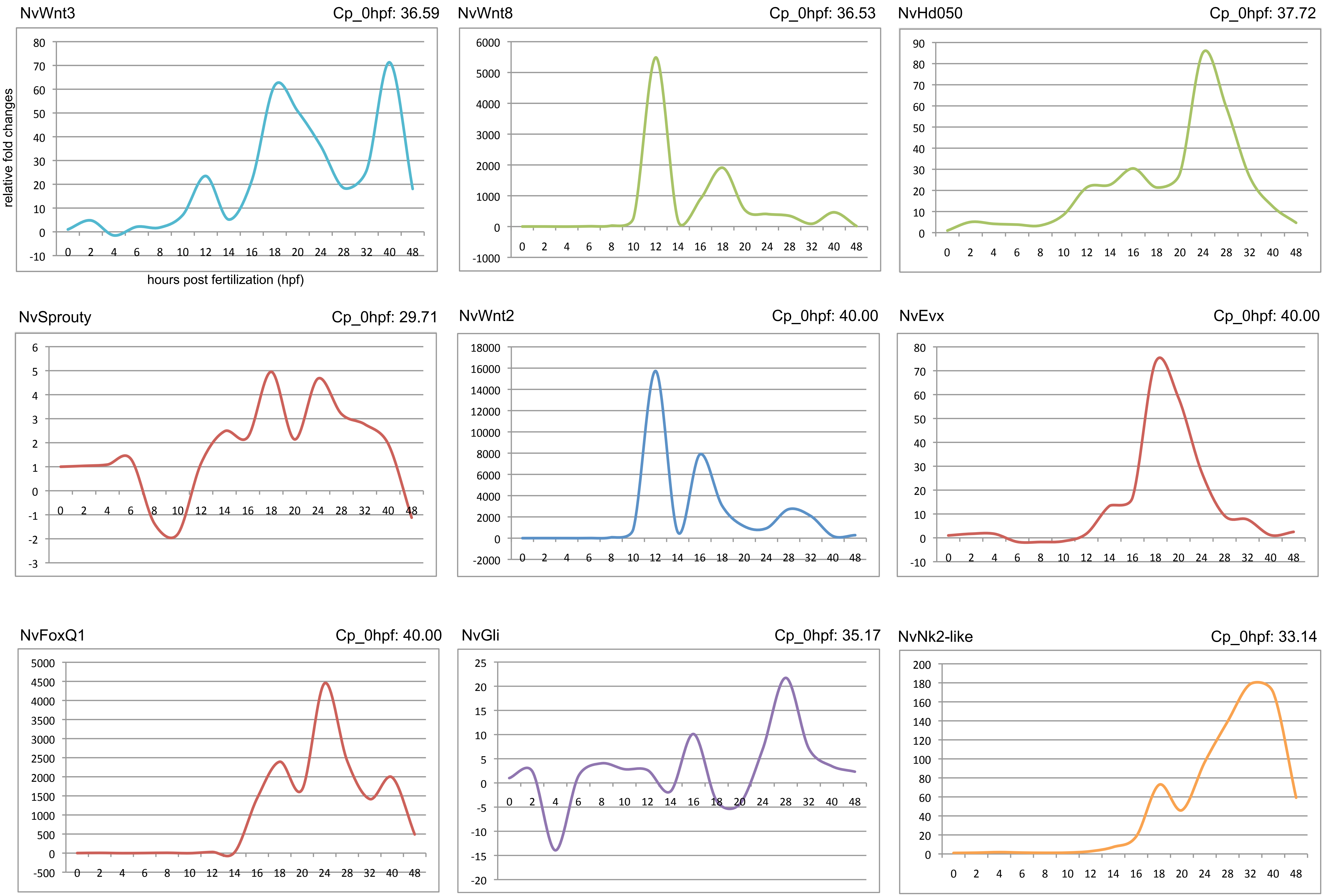
NvWnt3
Cp_0hpf: 36.59
relative fold changes
hours post fertilization (hpf)
NvWnt8
Cp_0hpf: 36.53
NvHd050
Cp_0hpf: 37.72
NvSprouty
Cp_0hpf: 29.71
NvWnt2
Cp_0hpf: 40.00
NvEvx
Cp_0hpf: 40.00
NvFoxQ1
Cp_0hpf: 40.00
NvGli
Cp_0hpf: 35.17
NvNk2-like
Cp_0hpf: 33.14

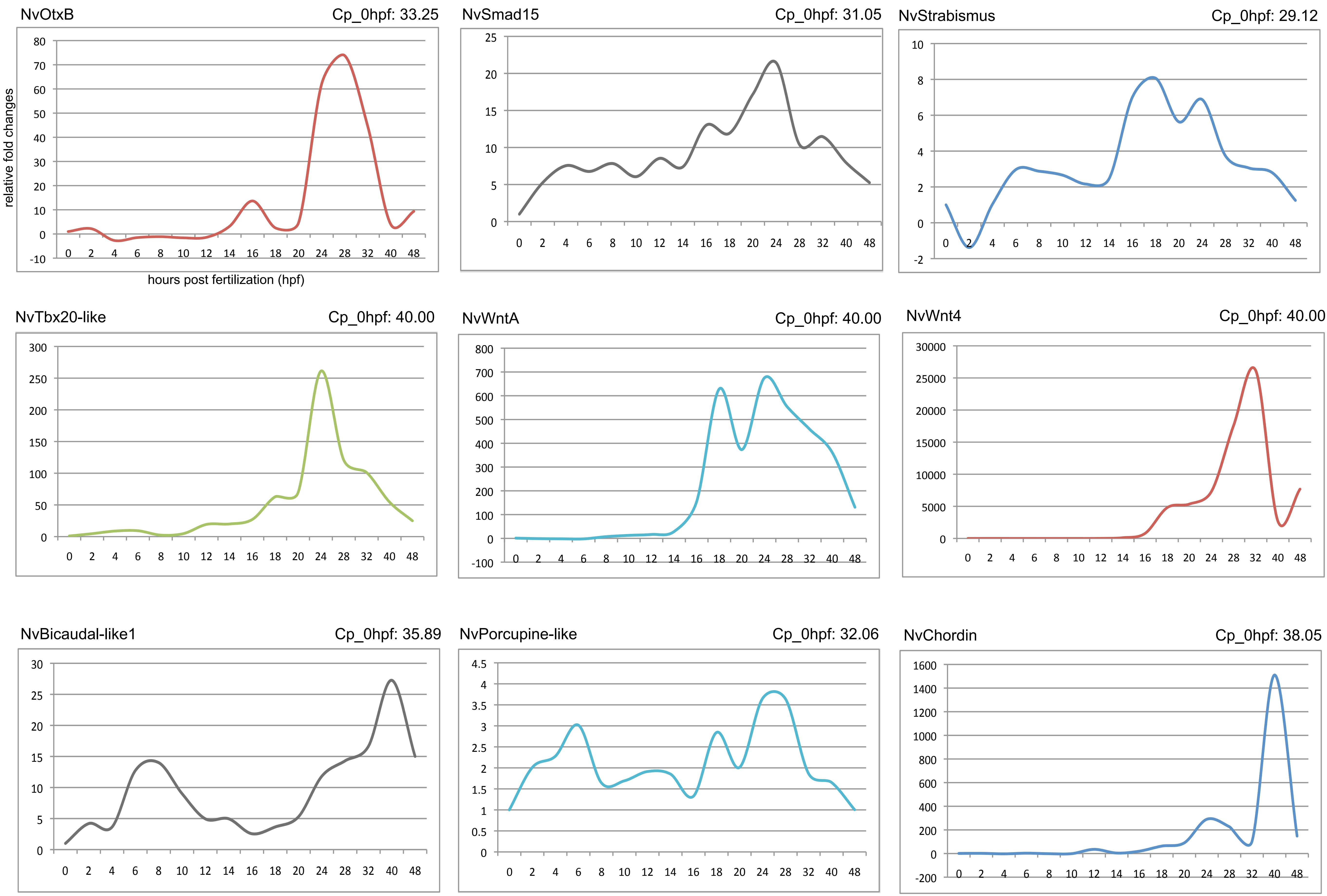

NvOtxB
Cp_0hpf: 33.25
relative fold changes
hours post fertilization (hpf)
NvSmad15
Cp_0hpf: 31.05
NvStrabismus
Cp_0hpf: 29.12
NvTbx20-like
Cp_0hpf: 40.00
NvWntA
Cp_0hpf: 40.00
NvWnt4
Cp_0hpf: 40.00
NvBicaudal-like1
Cp_0hpf: 35.89
NvPorcupine-like
Cp_0hpf: 32.06
NvChordin
Cp_0hpf: 38.05
0 2 4 6 8 10 12 14 16 18 20 24 28 32 40 48

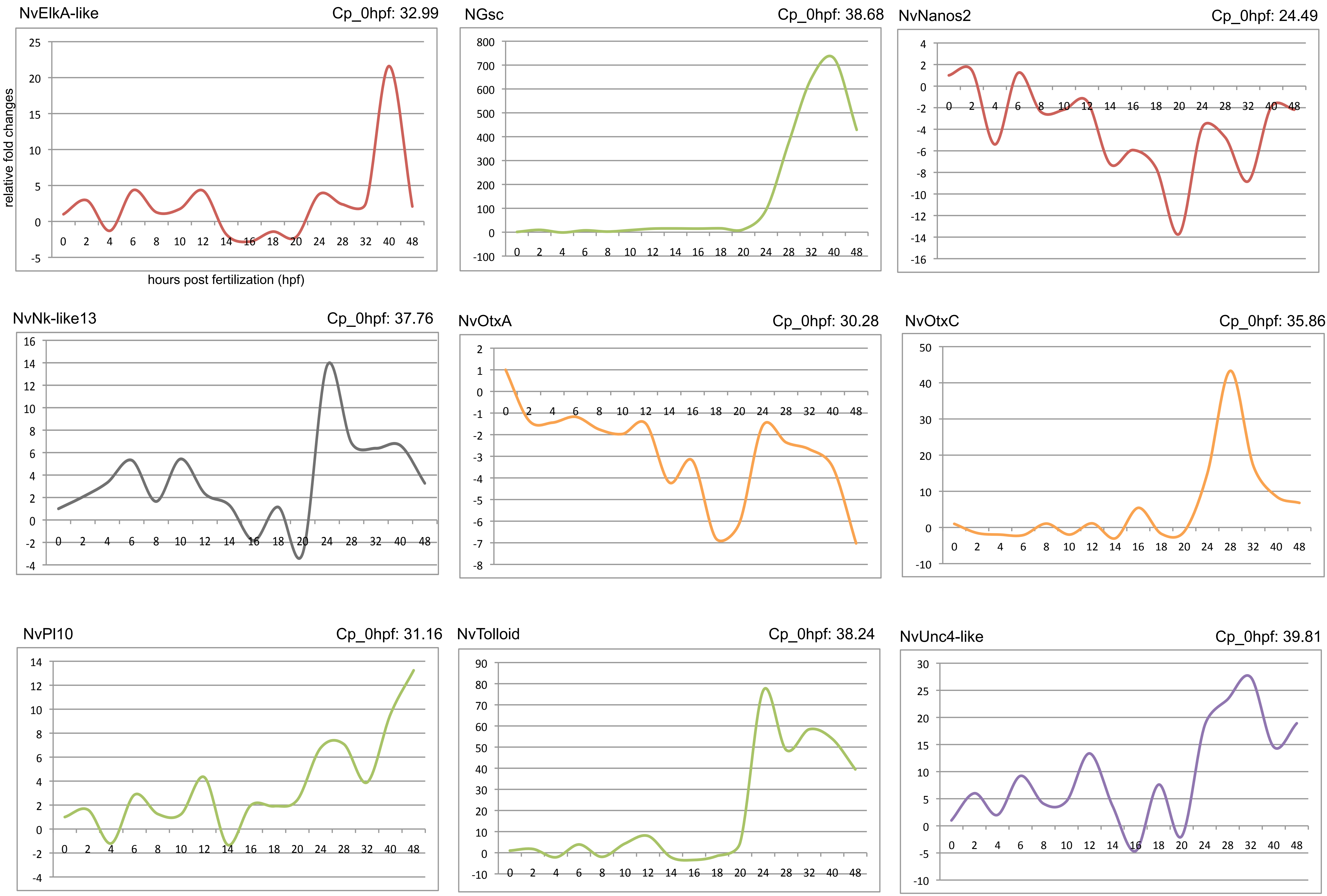
NvElkA-like
Cp_0hpf: 32.99
relative fold changes
hours post fertilization (hpf)
NGsc
Cp_0hpf: 38.68
NvNanos2
Cp_0hpf: 24.49
NvNk-like13
Cp_0hpf: 37.76
NvOtxA
Cp_0hpf: 30.28
NvOtxC
Cp_0hpf: 35.86
NvPl10
Cp_0hpf: 31.16
NvTolloid
Cp_0hpf: 38.24
NvUnc4-like
Cp_0hpf: 39.81
0 2 4 6 8 10 12 14 16 18 20 24 28 32 40 48

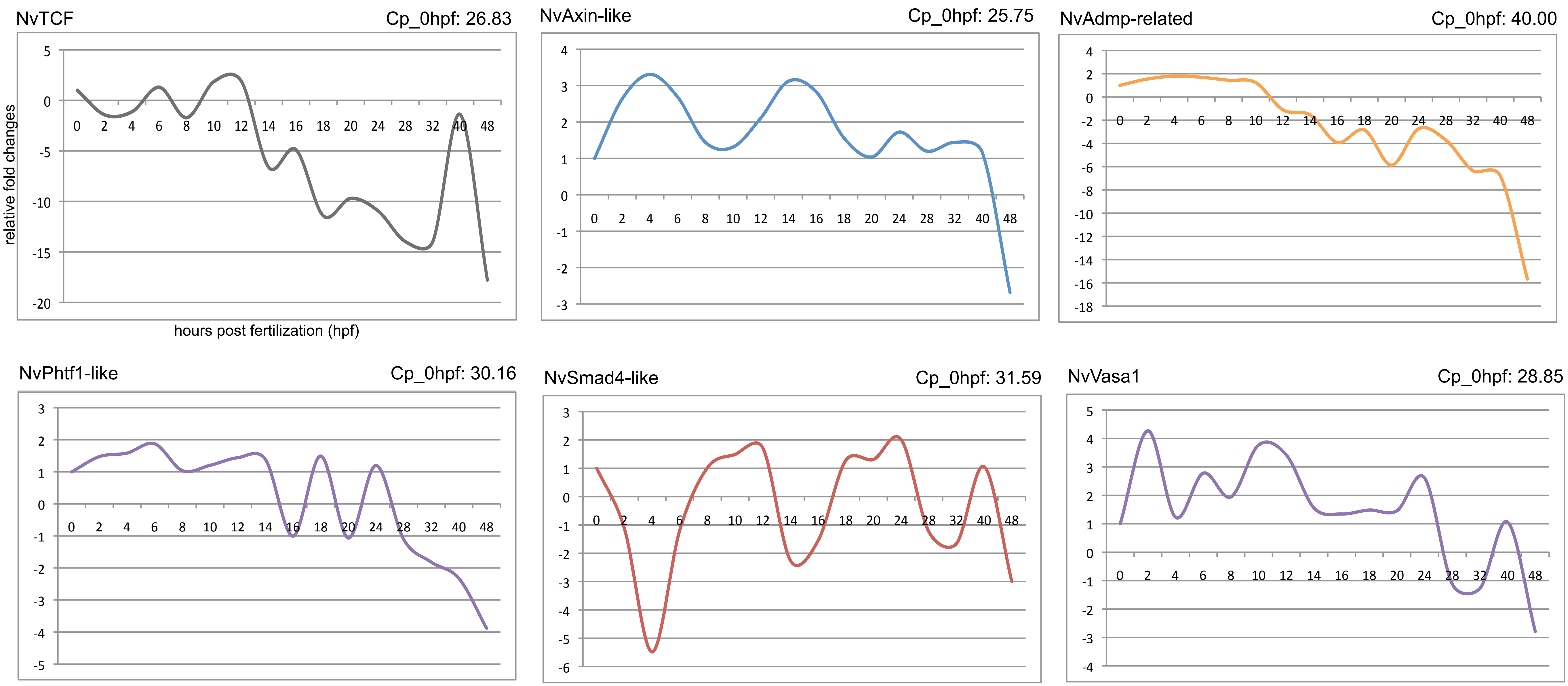
NvTCF
Cp_0hpf: 26.83
relative fold changes
5 0 -5 -10 -15 -20
0 2 4 6 8 10 12 14 16 18 20 24 28 32 40 48
hours post fertilization (hpf)
NvAxin-like
Cp_0hpf: 25.75
4 3 2 1 0 -1 -2 -3
0 2 4 6 8 10 12 14 16 18 20 24 28 32 40 48
NvAdmp-related
Cp_0hpf: 40.00
4 2 0 -2 -4 -6 -8 -10 -12 -14 -16 -18
0 2 4 6 8 10 12 14 16 18 20 24 28 32 40 48
NvPhtf1-like
Cp_0hpf: 30.16
3 2 1 0 -1 -2 -3 -4 -5
0 2 4 6 8 10 12 14 16 18 20 24 28 32 40 48
NvSmad4-like
Cp_0hpf: 31.59
3 2 1 0 -1 -2 -3 -4 -5 -6
0 2 4 6 8 10 12 14 16 18 20 24 28 32 40 48
NvVasa1
Cp_0hpf: 28.85
5 4 3 2 1 0 -1 -2 -3 -4
0 2 4 6 8 10 12 14 16 18 20 24 28 32 40 48
