# Supplementary material for: A Framework for the Establishment of a Cnidarian Gene Regulatory Network for “Endomesoderm” Specification: The Inputs of ß-Catenin/TCF Signaling
Source: PLoS Genet. 2012 Dec 27;8(12):e1003164. doi: 10.1371/journal.pgen.1003164 (PMC3531958; doi:10.1371/journal.pgen.1003164)
Supplement: Figure S6 — Gene expression analyzed by qPCR (additional genes). (A) Summary and (B) charts of high-density gene expression profiles for all genes not expressed (or undetected) in the animal hemisphere at the blastula stage (24 hpf) analyzed in this study. Y-axis indicates the relative fold change compared to unfertilized eggs. X-axis indicates developmental time in hours post fertilization. Gene names as indicated in the top left corner and the Cp value in unfertilized eggs is indicated in the top right corner of each panel that was used to determine the presence of maternal transcripts in Figure S6A (Cp>34.00). Cp corresponds to the crossing point (also known as Ct (cycle threshold) value). (PDF) [file pgen.1003164.s006.pdf]

Supplementary Figure 6

A gene	maternal (Cp)	zygotic UP	expression @ 24hpf
**hd043**	no (40.00)	8-10	nd
**hd032**	no (40.00)	8-10	nd
**moxC**	no (40.00)	8-10	nd
**activin**	no (40.00)	10-12	nd
**fgfA1**	no (40.00)	10-12	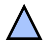
**foxA/B-like**	no (40.00)	10-12	nd
**hes3**	no (35.02)	10-12	ecto/sp
**twist**	no (40.00)	10-12	nd
**wnt1**	no (37.36)	10-12	nd
**wnt11**	no (40.00)	10-12	nd
**wnt16**	no (40.00)	10-12	nd
**follistatin-like**	**yes (32.89)**	14-16	nd
**hd017**	**yes (27.56)**	14-16	nd
**moxD**	no (38.76)	14-16	nd
**msxB**	no (36.67)	14-16	nd
**repo**	no (34.00)	14-16	nd
**gata**	**yes (33.59)**	16-18	ecto/sp
**tbx15-like**	no (38.71)	20-24	nd
**fgf8/17-like**	**yes (33.24)**	20-24	nd

nd not determined
ecto/sp salt & pepper expression (individual cells) in the ectoderm

B

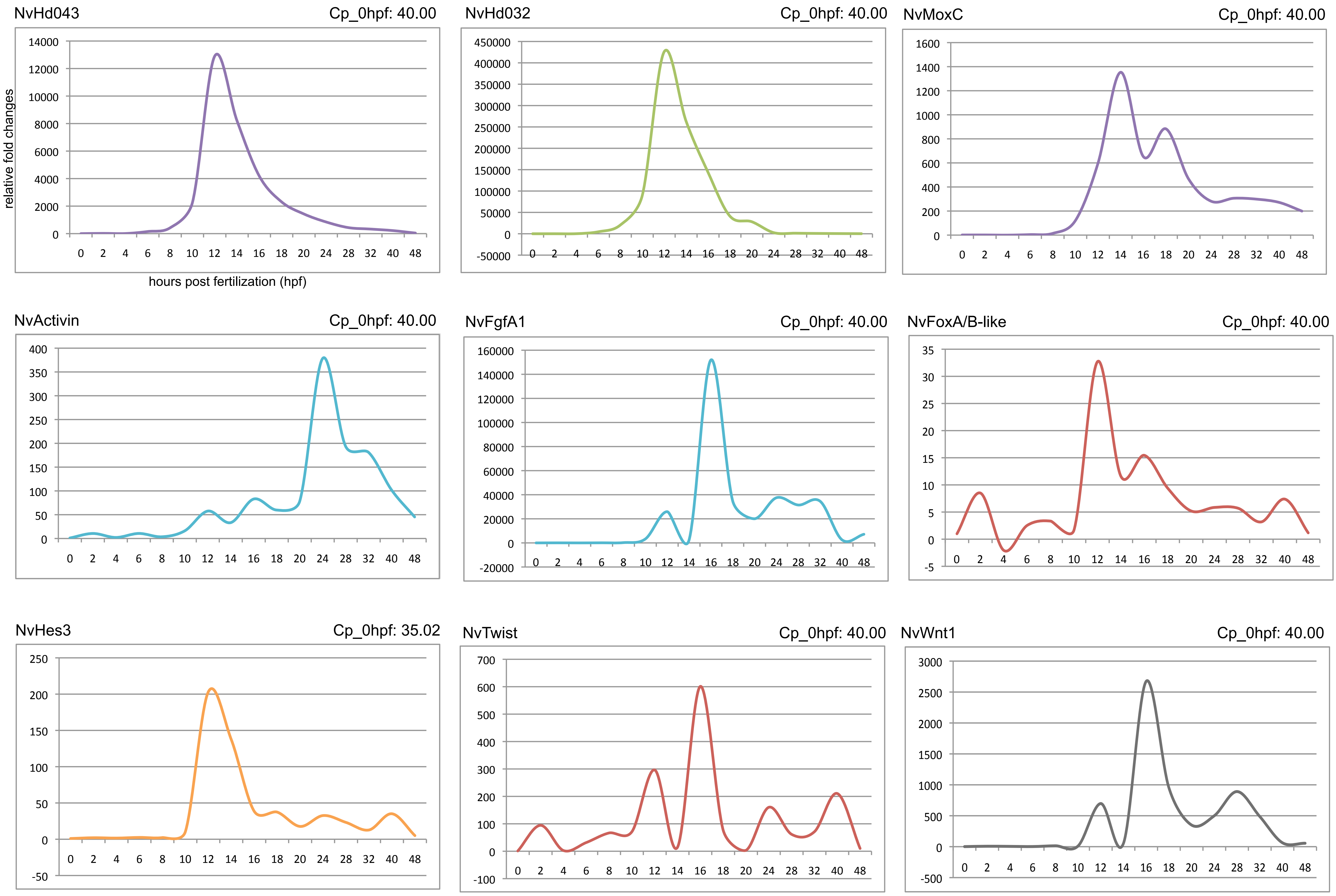

NvHd043
Cp_0hpf: 40.00
relative fold changes
hours post fertilization (hpf)
NvHd032
Cp_0hpf: 40.00
NvMoxC
Cp_0hpf: 40.00
NvActivin
Cp_0hpf: 40.00
NvFgfA1
Cp_0hpf: 40.00
NvFoxA/B-like
Cp_0hpf: 40.00
NvHes3
Cp_0hpf: 35.02
NvTwist
Cp_0hpf: 40.00
NvWnt1
Cp_0hpf: 40.00

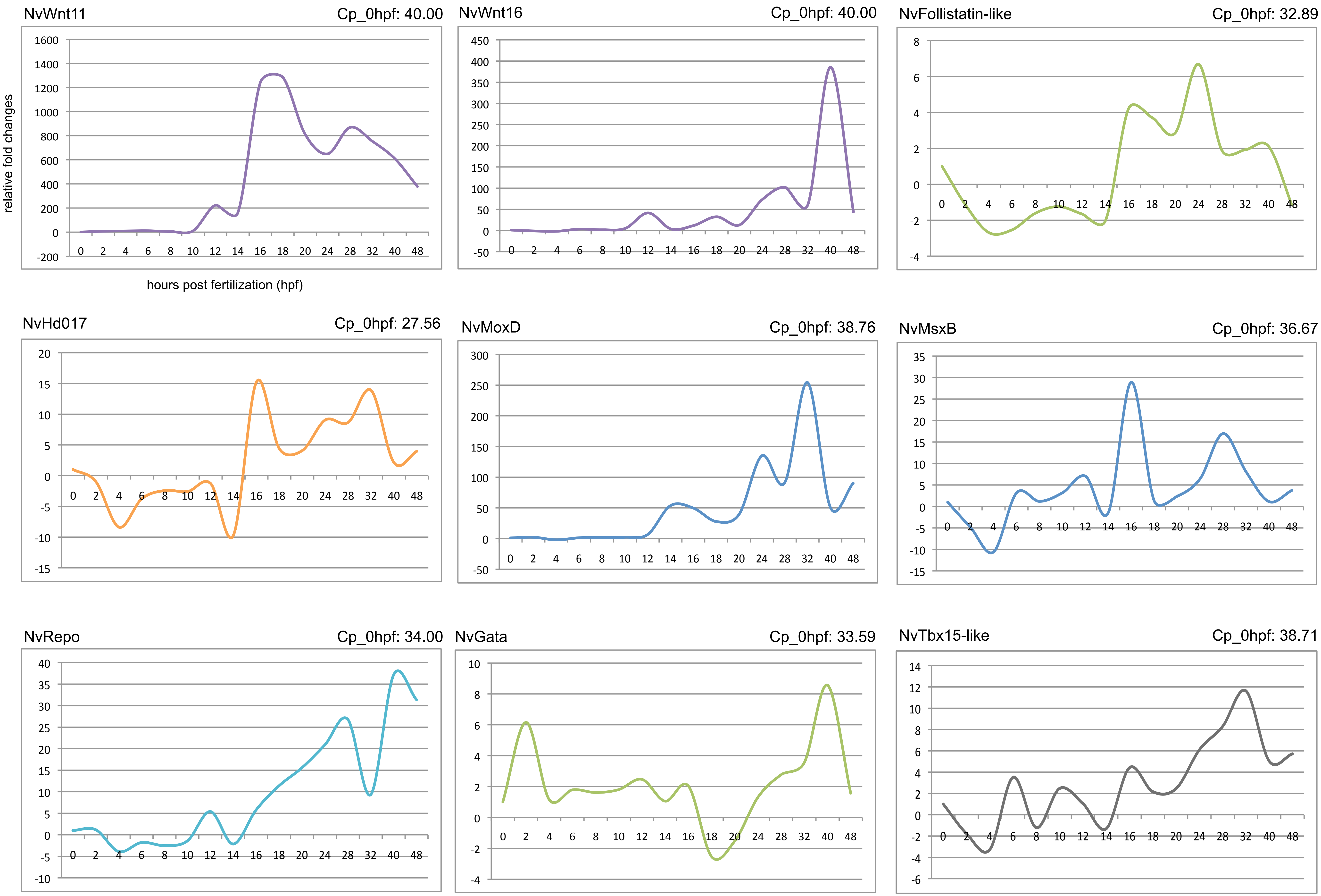

NvWnt11
Cp_0hpf: 40.00
NvWnt16
Cp_0hpf: 40.00
NvFollistatin-like
Cp_0hpf: 32.89
relative fold changes
hours post fertilization (hpf)
NvHd017
Cp_0hpf: 27.56
NvMoxD
Cp_0hpf: 38.76
NvMsxB
Cp_0hpf: 36.67
NvRepo
Cp_0hpf: 34.00
NvGata
Cp_0hpf: 33.59
NvTbx15-like
Cp_0hpf: 38.71
0 2 4 6 8 10 12 14 16 18 20 24 28 32 40 48

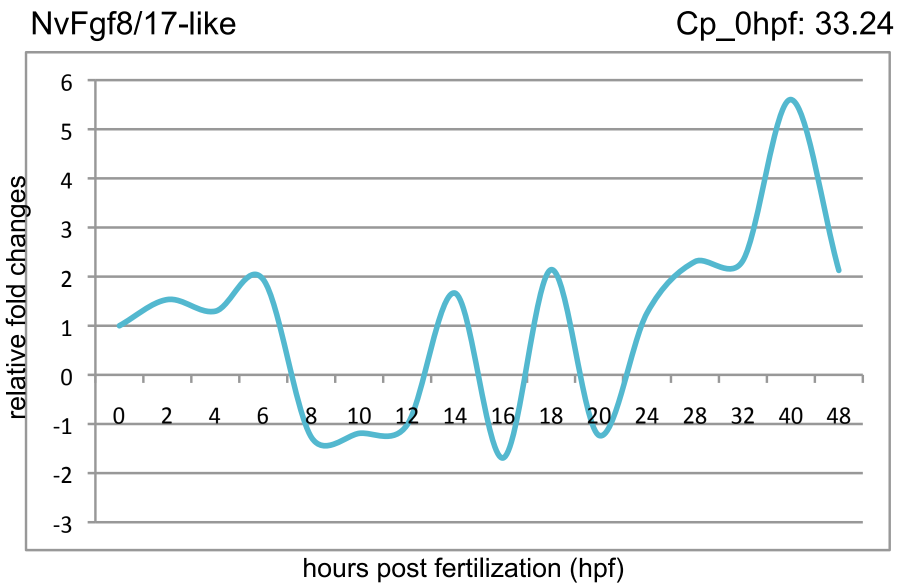

NvFgf8/17-like
Cp_0hpf: 33.24
relative fold changes
6
5
4
3
2
1
0
-1
-2
-3
0 2 4 6 8 10 12 14 16 18 20 24 28 32 40 48
hours post fertilization (hpf)
