# Supplementary material for: A Framework for the Establishment of a Cnidarian Gene Regulatory Network for “Endomesoderm” Specification: The Inputs of ß-Catenin/TCF Signaling
Source: PLoS Genet. 2012 Dec 27;8(12):e1003164. doi: 10.1371/journal.pgen.1003164 (PMC3531958; doi:10.1371/journal.pgen.1003164)

Supplementary Figure 8

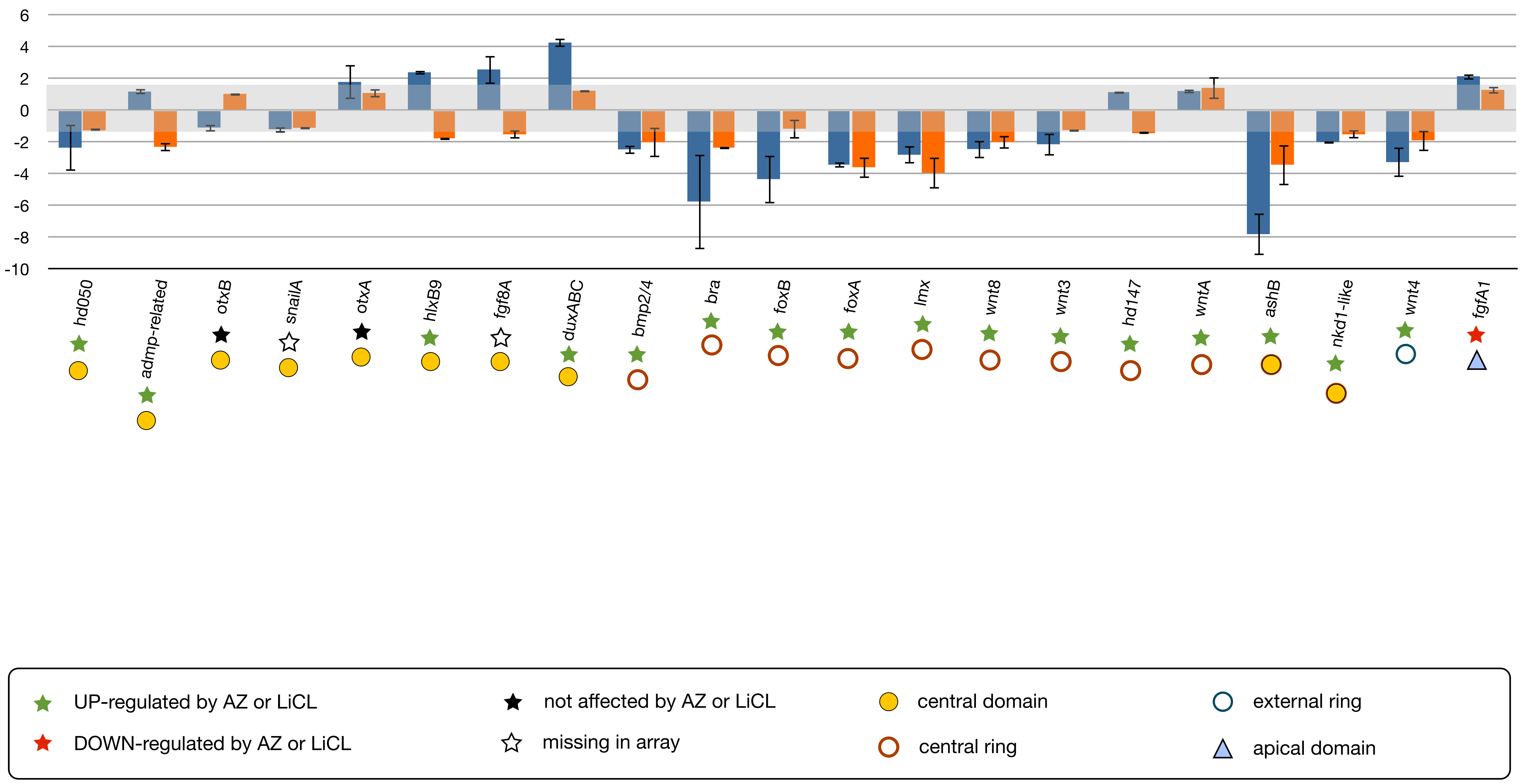

Supplement: Figure S8 — Comparative analysis of the molecular effects of MoTcf_trans and Nv-dnTcf:Venus by qPCR. Comparison of the effects on transcript levels after Nv-dntcf:Venus (blue) or MoTcf_trans (orange) injection showing an overall similar effect. (PDF) [file pgen.1003164.s008.pdf]
